# Concise communication title: wide adoption of rapid molecular detection of antimicrobial resistance markers for use with blood cultures: implications for national surveillance of antimicrobial resistance

**DOI:** 10.1017/ice.2025.67

**Published:** 2026-01

**Authors:** Hsiu Wu, Rupert England, Xueqing Huang, Joseph Lutgring, John Spinosa, Virgie Fields, Amy Webb, Marissa McMeen, Andrea Benin

**Affiliations:** 1 Division of Healthcare Quality Promotion, CDC, Atlanta, GA, USA; 2 Oak Ridge Institute for Science and Education, Oak Ridge, TN, USA; 3 Leidos, Reston, VA, USA; 4 Lantana Consulting Group, East Thetford, VT, USA

## Abstract

Rapid molecular testing for antimicrobial resistance (AR) provides an indication of resistance faster than phenotypic antimicrobial susceptibility testing. We summarize the adoption of molecular testing for AR among US acute care hospitals and discuss the potential impact on National Healthcare Safety Network’s surveillance for AR.

## Introduction

Antimicrobial resistance (AR) is a global public health issue.^1,2^ Surveillance for AR among healthcare facilities, including antimicrobial susceptibility testing (AST) data from pathogens recovered from clinical specimens, can inform prevention strategies and infection control responses. Phenotypic AST is a dependable source for AR surveillance due to the broad adoption of testing, interpretation, and reporting standards by clinical laboratories.^3,4^ However, the rapid molecular detection of AR markers, especially the tests that are used with blood cultures, has become increasingly prevalent in clinical laboratories in recent years. Compared to phenotypic AST, rapid molecular detection can provide a faster indication of AR. However, the results of rapid molecular tests are not reported to clinicians in a consistent manner.^5^ This reporting variation could undermine the ability to compare and aggregate AR data across facilities. The National Healthcare Safety Network (NHSN) is the nation’s most widely used tracking system for healthcare-associated infections.^6^ NHSN’s AR surveillance currently receives mainly culture-based phenotypic AST data extracted from hospital laboratory information systems.^7^ NHSN collects data on facility characteristics and infection control, laboratory, and antibiotic stewardship practices through the NHSN Annual Hospital Survey. In 2023, NHSN added questions to the survey about how hospitals use rapid molecular AR detection for bacteremia and how those results are interpreted and reported to clinicians. We analyzed hospitals’ responses to these questions to assess the potential influence of molecular AR testing on NHSN’s AR surveillance.

## Methods

Using the 2023 NHSN Annual Hospital Survey database (survey questions as in the supplemental material), we analyzed the responses we received as of July 29, 2024 for questions 6, 7, and 8, which pertain to the use of rapid AR diagnostics for bacterial bloodstream infections. Hospitals using AR markers with blood cultures included those selected “Yes” for question 6 except for those who selected only T2Candida or T2Bacteria, which do not require culture or provide information about AR markers. In questions 7 and 8, practices applied to *mecA* in *Staphylococcus aureus* and CTX-M in *Escherichia coli* blood isolates were used to assess how rapid molecular testing was used with phenotypic testing. χ^2^ and Fisher exact tests were used to compare hospital characteristics, including facility type, number of bed groups, and teaching status, between hospitals that reported using rapid molecular detection of AR markers for bacteremia and hospitals that reported not using them. Variables that showed significant differences between the two groups (*P* < 0.05) entered a multivariable logistic regression model, which we used to identify hospital characteristics independently associated with the use of rapid molecular AR testing. All statistical tests were conducted using SAS version 9.4 software (SAS Institute Inc, Cary, North Carolina).

## Results

### Uptake of rapid molecular tests for AR marker detection for use with blood cultures

**Figure 1.**
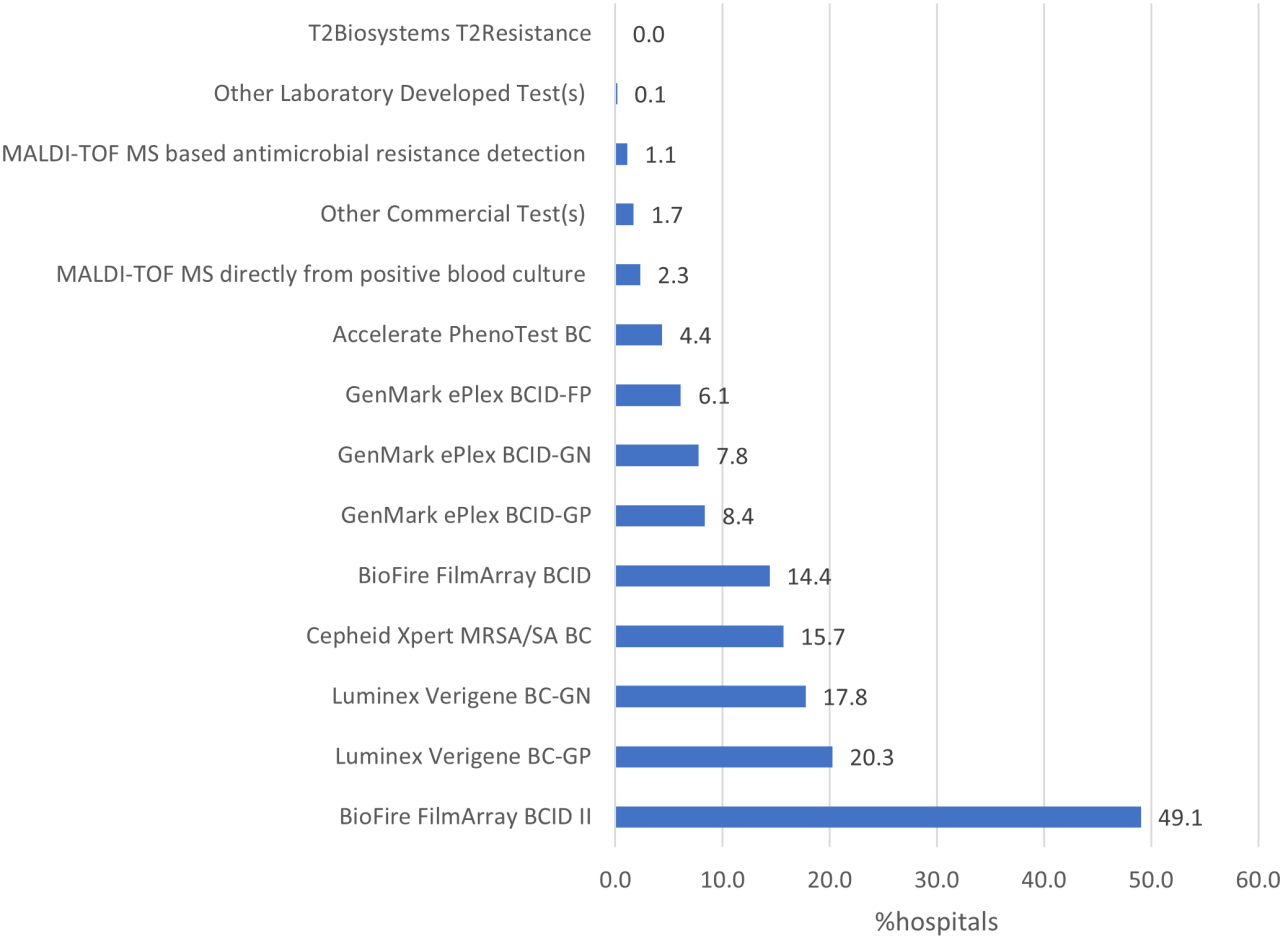
Percentage of hospitals using various panels of commercial or laboratory-developed tests for rapid molecular detection of antimicrobial resistance markers for use with bacterial blood cultures, National Healthcare Safety Network Annual Hospital Survey, 2023.

Of 5,316 hospitals eligible to complete the 2023 NHSN Annual Hospital survey, 5,301 (99.7%) responded. A total of 3,340 (63.0%) reported using at least one commercial or laboratory-developed test for rapid molecular detection of AR markers in bacterial bloodstream infections. The hospitals reporting using molecular tests for AR markers were more likely to be larger and teaching hospitals compared to those reporting no molecular tests for AR markers. Compared to general hospitals, critical access, military, and psychiatric hospitals were less likely, while orthopedic hospitals were more likely to use rapid molecular assays for AR markers detection in 2023 (Table 1). BioFire FilmArray BCID II (49.1%), Luminex Verigene BC-GP (20.3%), and Luminex Verigene BC-GN (17.8%) were the most used assays (Figure 1). Among hospitals reporting using molecular tests, 2,154 (64.5%) reported using one test, 809 (24.2%) reported using two tests, and 377 (11.3%) reported using 3–6 different tests.

**Table 1.** Characteristics of hospitals using commercial or laboratory-developed tests for rapid molecular detection of antimicrobial resistance markers in bloodstream infections, National Healthcare Safety Network Annual Hospital Survey, 2023

	Hospitals using rapid diagnostics for AR markers (n = 3342)	Hospitals not using rapid diagnostics for AR markers (n = 1959)	Odds Ratio [95% Confidence Limits]	P value
**Hospital type, n (%)**				<0.0001
General	2327 (69.67)	1085 (55.39)	Reference	
Critical access	656 (19.64)	645 (32.92)	0.75 [0.64–0.87]	
Veterans	94 (2.81)	31 (1.58)	1.48 [0.98–2.25]	
Children’s	85 (2.54)	26 (1.33)	1.46 [0.93–2.29]	
Surgical	54 (1.62)	53 (2.71)	0.77 [0.52–1.14]	
Psychiatric	45 (1.35)	74 (3.78)	0.35 [0.24–0.52]	
Orthopedic	30 (0.90)	6 (0.31)	3.28 [1.35–7.97]	
Military	18 (0.54)	27 (1.38)	0.42 [0.23–0.78]	
Oncology	12 (0.36)	8 (0.41)	0.71 [0.28–1.77]	
Women’s	11 (0.33)	2 (0.10)	2.47 [0.54–11.27]	
Women and children’s	8 (0.24)	2 (0.10)	1.68 [0.35–8.05]	
**Teaching status, n (%)**				<0.0001
Teaching	2515 (75.28)	1098 (56.05)	1.77 [1.56–2.02]	
Non-teaching	825 (24.70)	861 (43.95)	Reference	
**Hospital beds, n (%)**				<0.0001
≤100	1640 (49.10)	1343 (68.56)	Reference	
101–500	1485 (44.46)	580 (29.61)	1.52 [1.31–1.76]	
501–900	185 (5.54)	31 (1.58)	3.24 [2.17–4.83]	
>900	30 (0.90)	5 (0.26)	3.47 [1.32–9.10]	

### Laboratory interpretation and reporting of rapid AR molecular tests in association with phenotypic AST results

Among 3,182 hospitals performing *mecA* testing, in the scenario where *mecA* and *Staphylococcus aureus* are detected by molecular testing of a blood specimen, 3,063 (96.3%) reported performing phenotypic AST while 119 (3.6%) reported no phenotypic AST. Of those that performed phenotypic AST, 1,942 (63.4%) reported adding text to the phenotypic test results indicating results of the corresponding rapid molecular testing and/or its interpretation. Of hospitals performing both *mecA* and phenotypic AST, in the scenario of discordance between genotypic and phenotypic results, 2,293 (74.9%) reported performing further testing to identify the reason for the discordance and modifying the AST interpretations based on the further analysis; 535 (17.5%) reported no further testing and the results are reported separately for *mecA* and phenotypic AST. Lastly, 235 (7.7%) reported no further testing and the phenotypic result is overridden by the molecular test results if *mecA* is detected.

Among 2,833 hospitals performing *bla*_CTX-M_ (CTX-M) testing, in the scenario where CTX-M and *Escherichia coli* are detected by molecular testing in a blood specimen, 2,709 (95.6%) reported performing phenotypic AST while 124 (4.4%) reported no phenotypic AST. Of those that performed phenotypic AST, 1,694 (62.5%) reported adding text to the phenotypic test result indicating results of the corresponding rapid molecular testing and/or the interpretation. Of hospitals performing both CTX-M and phenotypic AST, in the scenario of discordant results, 2,013 (74.3%) reported performing further testing and modifying the AST interpretations based on the further analysis, 499 (18.4%) reported no further testing and the results are reported separately, and 197 (7.3%) reported no further testing and the phenotypic result is overridden by the molecular test results if CTX-M is detected.

## Discussion

Most U.S. hospitals report data to NHSN because of the requirements of the Centers for Medicare & Medicaid Services, and of the more than 5300 hospitals eligible to complete the NHSN survey in 2023, almost all submitted responses. Sixty-three percent reported using at least one commercial or laboratory-developed test for rapid molecular detection of AR markers with blood cultures. Use was more common in large and teaching hospitals. Most hospitals performed concurrent phenotypic AST; however, hospitals’ approaches to reporting AST and molecular test result interpretations and discordant results varied.

Our results contrast with those of Simner et al. in their survey of 96 clinical laboratories conducted between December 2020 and May 2021. They found that a much higher percentage of laboratories (93.8%) reported performing rapid diagnostics for AR markers on positive blood cultures. This difference is likely because of the characteristics of participating facilities; 46% of the 96 hospitals in the Simner study had >500 beds, compared with 5% of the 5301 hospitals that completed the NHSN survey.”

We observed substantial variability in hospitals’ practices for confirming results of rapid molecular testing for AR markers with phenotypic AST, and for conducting additional testing to resolve discordant results between molecular and phenotypic tests. This variability has the potential to bias national AR surveillance data collected and reported by NHSN. For example, although many molecular test manufacturers and the Clinical and Laboratory Standards Institute recommend confirming the results of rapid molecular tests for AR markers with phenotypic AST,^3,8^ approximately 4% of hospitals in our survey did not perform this testing. In such cases, incident AR detected by molecular tests may be overlooked by a surveillance system that only accepts phenotypic AST results. Additionally, 25% of hospitals did not conduct recommended^3^ additional testing to resolve discordant results; some of these hospitals reported discordant phenotypic and molecular test results separately, and others overriding phenotypic results with the positive molecular results, in which resistance genes with low or no expression may be included.^9^ These different practices could lead to differences in the ability to detect and report AR, potentially affecting NHSN’s ability to provide accurate national AR data for benchmarking and inter-facility comparisons. NHSN also faces a challenge of accounting for various frequencies, genes, and panels used across different hospitals in order to achieve fair benchmarking and inter-facility comparison.^4^

As rapid molecular AR tests become more widely adopted, it is necessary to integrate and standardize the reporting to clinicians as well as into national AR surveillance. NHSN team is working toward scaling AR surveillance module to capture and summarize molecular AR data for national surveillance.

## Supporting information

Wu et al. supplementary material Wu et al. supplementary material

## Supplementary material

For supplementary material/s referred to in this article, please visit https://doi.org/10.1017/ice.2025.67
