# Supplementary material for: Concise communication title: wide adoption of rapid molecular detection of antimicrobial resistance markers for use with blood cultures: implications for national surveillance of antimicrobial resistance
Source: Infect Control Hosp Epidemiol. 2025 Nov 6;47(1):94–7. doi: 10.1017/ice.2025.67 (PMC12780831; doi:10.1017/ice.2025.67)
Supplement: Wu et al. supplementary material [file S0899823X25000674sup001.pdf]

NATIONAL HEALTHCARE SAFETY NETWORK

Form Approved
OMB No. 0920-0666
Exp. Date: 12/31/2026
www.cdc.gov/nhsn

Patient Safety Component—Annual Hospital Survey

Instructions for this form are available at: http://www.cdc.gov/nhsn/forms/instr/57_103-TOI.pdf

*required for saving

Tracking #:

Facility ID:

*Survey Year:

Facility Characteristics (completed by Infection Preventionist)

*Ownership (check one):

☐ For profit ☐ Not for profit, including church ☐ Government

☐ Military ☐ Veterans Affairs ☐ Physician owned

If facility is a Hospital:

*Number of patient days:________

*Number of admissions:_________

For any Hospital:

*Is your hospital a teaching hospital for physician and/or physicians-in-training or nursing students? ☐ Yes ☐ No

If Yes, what type: ☐ Major ☐ Graduate ☐ Undergraduate

*Number of beds set up and staffed in the following location types (as defined by NHSN):

a. ICU (including adult, pediatric, and neonatal levels II/III, III or higher): ________________

b. All other inpatient locations: ________________

Facility Microbiology Laboratory Practices (completed with input from Microbiology Laboratory Lead)

*1. Does your facility have its own on-site laboratory that performs bacterial antimicrobial susceptibility testing? ☐ Yes ☐ No

a. If No, where is your facility's antimicrobial susceptibility testing performed? (check one)

☐ Affiliated medical center

☐ Commercial referral laboratory

☐ Other local/regional, non-affiliated reference laboratory

b. If Yes, do you also send out any antimicrobial susceptibility testing? (check one) ☐ Yes ☐ No

*2. For the following organisms, indicate which methods are used for:

Assurance of Confidentiality: The voluntarily provided information obtained in this surveillance system that would permit identification of any individual or institution is collected with a guarantee that it will be held in strict confidence, will be used only for the purposes stated, and will not otherwise be disclosed or released without the consent of the individual, or the institution in accordance with Sections 304, 306 and 308(d) of the Public Health Service Act (42 USC 242b, 242k, and 242m(d)).

Public reporting burden of this collection of information is estimated to average 135 minutes per response, including the time for reviewing instructions, searching existing data sources, gathering, and maintaining the data needed, and completing and reviewing the collection of information. An agency may not conduct or sponsor, and a person is not required to respond to a collection of information unless it displays a currently valid OMB control number. Send comments regarding this burden estimate or any other aspect of this collection of information, including suggestions for reducing this burden to CDC, Reports Clearance Officer, 1600 Clifton Rd., MS H-21, Atlanta, GA 30333, ATTN: PRA (0920-0666).

(1) Primary susceptibility testing and
(2) Secondary, supplemental, or confirmatory testing (if performed).

Facility Microbiology Laboratory Practices (continued)

If your laboratory does not perform susceptibility testing, indicate the methods used at the outside laboratory.

Use the testing codes listed below the table.

Pathogen	(1) Primary	(2) Secondary	Comments
Enterobacterales	____________	____________	____________
Pseudomonas aeruginosa	____________	____________	____________
Acinetobacter baumanni complex	____________	____________	____________

1 = Kirby-Bauer disk diffusion	4 = Sensititre	7 = Agar dilution method
2 = Vitek (Legacy)	5.1 = MicroScan WalkAway	10 = Gradient Dilution Strip (for example E test)
2.1 = Vitek 2	5.2 = MicroScan autoSCAN	13 = Other (describe in Comments section)
3.1 = BD Phoenix	6 = Other broth microdilution method	

*3. Does either primary or secondary/supplemental antimicrobial susceptibility testing (AST) include the following (check all that apply):

**Drug**	***Organism tested:***		
	Enterobacterales	***Pseudomonas aeruginosa***	***Acinetobacter baumanni***
Cefiderocol	☐	☐	☐
Ceftazidime-Avibactam	☐	☐	☐
Ceftolozane-Tazobactam	☐	☐	☐
Colistin	☐	☐	☐

Assurance of Confidentiality: The voluntarily provided information obtained in this surveillance system that would permit identification of any individual or institution is collected with a guarantee that it will be held in strict confidence, will be used only for the purposes stated, and will not otherwise be disclosed or released without the consent of the individual, or the institution in accordance with Sections 304, 306 and 308(d) of the Public Health Service Act (42 USC 242b, 242k, and 242m(d)).
CDC 57.103 (Front) Rev. 15, v12.0

Public reporting burden of this collection of information is estimated to average 135 minutes per response, including the time for reviewing instructions, searching existing data sources, gathering, and maintaining the data needed, and completing and reviewing the collection of information. An agency may not conduct or sponsor, and a person is not required to respond to a collection of information unless it displays a currently valid OMB control number. Send comments regarding this burden estimate or any other aspect of this collection of information, including suggestions for reducing this burden to CDC, Reports Clearance Officer, 1600 Clifton Rd., MS H-21, Atlanta, GA 30333, ATTN: PRA (0920-0666).

Delafloxacin	☐	☐	☐
Eravacycline	☐	☐	☐
Imipenem-Relebactam	☐	☐	☐
Meropenem-Vaborbactam	☐	☐	☐

Assurance of Confidentiality: The voluntarily provided information obtained in this surveillance system that would permit identification of any individual or institution is collected with a guarantee that it will be held in strict confidence, will be used only for the purposes stated, and will not otherwise be disclosed or released without the consent of the individual, or the institution in accordance with Sections 304, 306 and 308(d) of the Public Health Service Act (42 USC 242b, 242k, and 242m(d)).

Public reporting burden of this collection of information is estimated to average 135 minutes per response, including the time for reviewing instructions, searching existing data sources, gathering, and maintaining the data needed, and completing and reviewing the collection of information. An agency may not conduct or sponsor, and a person is not required to respond to a collection of information unless it displays a currently valid OMB control number. Send comments regarding this burden estimate or any other aspect of this collection of information, including suggestions for reducing this burden to CDC, Reports Clearance Officer, 1600 Clifton Rd., MS H-21, Atlanta, GA 30333, ATTN: PRA (0920-0666).

Facility Microbiology Laboratory Practices (continued)

*4. Has the laboratory implemented revised breakpoints recommended by CLSI for the following:

a. Third Generation Cephalosporin and monobactam (i.e. aztreonam) breakpoints for *Enterobacterales* in 2010	☐ Yes ☐ No
b. Carbapenem breakpoints for *Enterobacterales* in 2010	☐ Yes ☐ No
c. Ertapenem breakpoints for *Enterobacterales* in 2012	☐ Yes ☐ No
d. Carbapenem breakpoints for *Pseudomonas aeruginosa* in 2012	☐ Yes ☐ No
e. Fluroquinolone breakpoints for *Pseudomonas aeruginosa* in 2019	☐ Yes ☐ No
f. Fluroquinolone breakpoints for *Enterobacterales* in 2019	☐ Yes ☐ No

*5. Does the laboratory test bacterial isolates for presence of carbapenemase? (this does not include automated testing instrument expert rules) ☐ Yes ☐ No

5a. If Yes, indicate what is done if carbapenemase production is detected: (check one)

- ☐ Change susceptible carbapenem results to resistant
- ☐ Report carbapenem MIC results without an interpretation
- ☐ No changes are made in the interpretation of carbapenems, the test is used for epidemiological or infection control practices

5b. If Yes, which test is routinely performed to detect carbapenemase: (check all that apply)

☐ NAAT (for example, PCR)	☐ MLB Screen
☐ Modified Hodge Test	☐ Carba NP
☐ mCIM/CIM	☐ Rapid CARB Blue
☐ E test	☐ CARBA 5
☐ Cepheid, BioFire, Verigene, Genmark, etc	☐ Other (specify):_____________

5c. If Yes, which of the following are routinely tested for the presence of carbapenemases: (check all that apply)

☐ *Enterobacterales* spp. ☐ *Pseudomonas aeruginosa* ☐ *Acinetobacter baumannii*

*6. Does your facility use commercial or laboratory developed tests for rapid molecular detection of antimicrobial resistance markers in bacterial bloodstream infections? Examples of commercially available systems include BioFire FilmArray, Luminex Verigene, etc.

☐ Yes

☐ No [If checked, skip questions 7 and 8]

Assurance of Confidentiality: The voluntarily provided information obtained in this surveillance system that would permit identification of any individual or institution is collected with a guarantee that it will be held in strict confidence, will be used only for the purposes stated, and will not otherwise be disclosed or released without the consent of the individual, or the institution in accordance with Sections 304, 306 and 308(d) of the Public Health Service Act (42 USC 242b, 242k, and 242m(d)).
CDC 57.103 (Front) Rev. 15, v12.0

Public reporting burden of this collection of information is estimated to average 135 minutes per response, including the time for reviewing instructions, searching existing data sources, gathering, and maintaining the data needed, and completing and reviewing the collection of information. An agency may not conduct or sponsor, and a person is not required to respond to a collection of information unless it displays a currently valid OMB control number. Send comments regarding this burden estimate or any other aspect of this collection of information, including suggestions for reducing this burden to CDC, Reports Clearance Officer, 1600 Clifton Rd., MS H-21, Atlanta, GA 30333, ATTN: PRA (0920-0666).

Facility Microbiology Laboratory Practices (continued)

6a. If Yes, which test panel(s) does your facility use? (check all that apply)

☐ Accelerate PhenoTest BC ☐ BioFire FilmArray BCID ☐ BioFire FilmArray BCID II
☐ Cepheid Xpert MRSA/SA BC ☐ GenMark ePlex BCID-GP ☐ GenMark ePlex BCID-GN
☐ GenMark ePlex BCID-FP ☐ Luminex Verigene BC-GP ☐ Luminex Verigene BC-GN
☐ MALDI-TOF MS directly from positive blood culture (e.g., SepsiTyper)
☐ MALDI-TOF MS based antimicrobial resistance detection
☐ T2Biosystems T2Bacteria ☐ T2Biosystems T2Candida ☐ T2Biosystems T2Resistance
☐ Other Commercial Test(s) (Leave Comment) ___________________________________
☐ Other Laboratory Developed Test(s) (Leave Comment) ___________________________

*7. In a scenario where the *mecA* resistance marker and *Staphylococcus aureus* are detected by rapid molecular testing in a blood specimen, select the procedure(s) your facility conducts. (check one)

☐ Our laboratory does not perform *mecA* testing using rapid molecular methods. [If checked, skip question 7a.]

☐ Culture based phenotypic antimicrobial susceptibility testing is not performed. [If checked, skip question 7a.]

☐ Culture based phenotypic antimicrobial susceptibility testing is performed. A text indicating results of the corresponding rapid molecular testing and/or the interpretation of the rapid molecular testing result is added to the phenotypic test result.

☐ Culture based phenotypic antimicrobial susceptibility testing is performed. No text indicating corresponding rapid molecular testing and/or interpretation is added.

7a. If both rapid molecular and culture based phenotypic antimicrobial susceptibility testing are performed for a blood specimen to detect drug resistance in *Staphylococcus aureus*, and discordance is found between their results, how are results reported? (check one)

☐ Further testing is not pursued. Results are reported separately.
☐ Further testing is not pursued. The phenotypic result is overridden by the rapid molecular test result when an antimicrobial resistance marker is detected.
☐ Further testing is performed to identify the reason for the discordance. Results are modified based on the further analysis.

*8. In a scenario where the $bla_{CTX\text{-}M}$ (CTX-M) resistance marker and *Escherichia coli* are detected by rapid molecular testing in a blood specimen, select the procedure(s) your facility conducts. (check one)

☐ Our laboratory does not perform $bla_{CTX\text{-}M}$ (CTX-M) testing using rapid molecular methods. [If checked, skip question 8a.]

☐ Culture based phenotypic antimicrobial susceptibility testing is not performed. [If checked, skip question 8a.]

Assurance of Confidentiality: The voluntarily provided information obtained in this surveillance system that would permit identification of any individual or institution is collected with a guarantee that it will be held in strict confidence, will be used only for the purposes stated, and will not otherwise be disclosed or released without the consent of the individual, or the institution in accordance with Sections 304, 306 and 308(d) of the Public Health Service Act (42 USC 242b, 242k, and 242m(d)).
CDC 57.103 (Front) Rev. 15, v12.0

Public reporting burden of this collection of information is estimated to average 135 minutes per response, including the time for reviewing instructions, searching existing data sources, gathering, and maintaining the data needed, and completing and reviewing the collection of information. An agency may not conduct or sponsor, and a person is not required to respond to a collection of information unless it displays a currently valid OMB control number. Send comments regarding this burden estimate or any other aspect of this collection of information, including suggestions for reducing this burden to CDC, Reports Clearance Officer, 1600 Clifton Rd., MS H-21, Atlanta, GA 30333, ATTN: PRA (0920-0666).

☐ Culture based phenotypic antimicrobial susceptibility testing is performed. A text indicating results of the corresponding rapid molecular testing and/or the interpretation of the rapid molecular testing result is added to the phenotypic test result.

☐ Culture based phenotypic antimicrobial susceptibility testing is performed. No text indicating corresponding rapid molecular testing and/or interpretation is added.

Facility Microbiology Laboratory Practices (continued)

8a. If both rapid molecular and culture based phenotypic antimicrobial susceptibility testing are performed for a blood specimen to detect drug resistance in *Escherichia coli* and discordance is found between their results, how are results reported? (check one)

☐ Further testing is not pursued. Results are reported separately.

☐ Further testing is not pursued. The phenotypic result is overridden by the rapid molecular test result when an antimicrobial resistance marker is detected.

☐ Further testing is performed to identify the reason for the discordance. Results are modified based on the further analysis.

*9. Does your facility perform extended-spectrum beta-lactamase (ESBL) testing for *E. coli, Klebsiella pneumoniae, Klebsiella oxytoca,* or *Proteus mirabilis* routinely or using a testing algorithm? ☐ Yes ☐ No

9a. If Yes, indicate what is done if ESBL is detected: (check one)

☐ Change susceptible Cefotaxime/Ceftriaxone/Cefepime results to resistant

☐ No changes are made in the interpretation of cephalosporins with a note of ESBL

☐ Suppress cephalosporin susceptibility results

*10. Where is yeast identification performed for specimens collected at your facility? (check one)

☐ On-site laboratory

☐ Affiliated medical center

☐ Commercial referral laboratory

☐ Other local/regional, non-affiliated reference laboratory

☐ Yeast identification not available (specifically, yeast identification is not performed onsite or at any affiliate/commercial/other laboratory) [If checked, skip questions 11-15]

Answer questions 11-15 for the laboratory that <u>performs yeast identification for your facility:</u>

*11. Which of the following methods are used for yeast identification? (check all that apply)

☐ MALDI-TOF MS System (Vitek MS) ☐ MicroScan

Assurance of Confidentiality: The voluntarily provided information obtained in this surveillance system that would permit identification of any individual or institution is collected with a guarantee that it will be held in strict confidence, will be used only for the purposes stated, and will not otherwise be disclosed or released without the consent of the individual, or the institution in accordance with Sections 304, 306 and 308(d) of the Public Health Service Act (42 USC 242b, 242k, and 242m(d)).
CDC 57.103 (Front) Rev. 15, v12.0

Public reporting burden of this collection of information is estimated to average 135 minutes per response, including the time for reviewing instructions, searching existing data sources, gathering, and maintaining the data needed, and completing and reviewing the collection of information. An agency may not conduct or sponsor, and a person is not required to respond to a collection of information unless it displays a currently valid OMB control number. Send comments regarding this burden estimate or any other aspect of this collection of information, including suggestions for reducing this burden to CDC, Reports Clearance Officer, 1600 Clifton Rd., MS H-21, Atlanta, GA 30333, ATTN: PRA (0920-0666).

☐ MALDI-TOF MS System (Bruker Biotyper)
☐ Vitek-2
☐ BD Phoenix
☐ Non-automated Manual Kit (for example, API 20C, RapID, Germ Tube, PNA-FISH, etc.)
☐ DNA sequencing
☐ Other (specify): ______________________

*12. Does the laboratory routinely use chromogenic agar for the identification or differentiation of *Candida* isolates?

☐ Yes ☐ No ☐ Unknown

*13. *Candida* isolated from which of the following body sites are usually fully identified to the species level? (check all that apply)

☐ Blood
☐ Other normally sterile body site (for example, CSF)
☐ Urine
☐ Respiratory
☐ Other (specify): ______________________
☐ None are fully identified to the species level

Facility Microbiology Laboratory Practices (continued)

*14. Does the laboratory employ any molecular tests to identify *Candida* from blood specimens?

☐ Yes ☐ No ☐ Unknown

14a. If yes, which molecular tests are used to identify *Candida* from blood specimens? (check all that apply)

☐ T2Candida Panel
☐ BioFire BCID
☐ GenMark ePlex BCID
☐ Other, specify: _________________
☐ Unknown

14b. If yes and you get a positive result, does this lab culture the blood to obtain an isolate?

☐ Yes, always
☐ Yes, with clinical order
☐ No
☐ Unknown

*15. Where is antifungal susceptibility testing (AFST) performed for specimens collected at your facility? (check one)

☐ On-site laboratory
☐ Affiliated medical center
☐ Commercial reference laboratory
☐ Other local/regional, non-affiliated reference laboratory
☐ AFST not available (specifically, AFST is not performed onsite or at any affiliate/commercial/other laboratory) [if selected, skip questions 16 -19]

Assurance of Confidentiality: The voluntarily provided information obtained in this surveillance system that would permit identification of any individual or institution is collected with a guarantee that it will be held in strict confidence, will be used only for the purposes stated, and will not otherwise be disclosed or released without the consent of the individual, or the institution in accordance with Sections 304, 306 and 308(d) of the Public Health Service Act (42 USC 242b, 242k, and 242m(d)).
CDC 57.103 (Front) Rev. 15, v12.0

Public reporting burden of this collection of information is estimated to average 135 minutes per response, including the time for reviewing instructions, searching existing data sources, gathering, and maintaining the data needed, and completing and reviewing the collection of information. An agency may not conduct or sponsor, and a person is not required to respond to a collection of information unless it displays a currently valid OMB control number. Send comments regarding this burden estimate or any other aspect of this collection of information, including suggestions for reducing this burden to CDC, Reports Clearance Officer, 1600 Clifton Rd., MS H-21, Atlanta, GA 30333, ATTN: PRA (0920-0666).

Answer questions 16-19 for the laboratory that performs AFST for your facility:

*16. What method is used for antifungal susceptibility testing (AFST), **excluding Amphotericin B?** (check all that apply)

☐ Broth microdilution with laboratory developed plates
☐ YeastOne (Thermo Scientific™ Sensititre™)
☐ Gradient diffusion (E test)
☐ Vitek (bioMerieux)
☐ Other (specify): ________________
☐ Unknown

*17.What method is used for antifungal susceptibility testing (AFST) of **Amphotericin B?** (check all that apply)

☐ Broth microdilution with laboratory developed plates
☐ YeastOne (Thermo Scientific™ Sensititre™)
☐ Gradient diffusion (E test)
☐ Vitek (bioMerieux)
☐ Other (specify): ________________
☐ Unknown

*18. AFST is performed for which of the following antifungal drugs? (check all that apply)

☐ Fluconazole
☐ Voriconazole
☐ Itraconazole
☐ Posaconazole
☐ Micafungin
☐ Anidulafungin
☐ Caspofungin
☐ Amphotericin B
☐ Flucytosine
☐ Other, specify: ________________
☐ Unknown

Facility Microbiology Laboratory Practices (continued)

*19. AFST is performed on fungal isolates in which of the following situations? (check only one box per row)

	Performed automatically	Performed with a clinician's order	Not performed	Unknown
Blood	☐	☐	☐	☐
Other normally sterile body site (for example, CSF)	☐	☐	☐	☐
Urine	☐	☐	☐	☐
Respiratory	☐	☐	☐	☐
Other (specify): _________	☐	☐	☐	☐

*20. Is this laboratory developing antibiograms or other reports to track susceptibility trends for *Candida* spp. isolates tested in this laboratory?

☐ Yes ☐ No ☐ Unknown

*21.What is the primary testing method for *C. difficile* used most often by your facility's laboratory or the outside laboratory where your facility's testing is performed? (check one)

☐ Enzyme immunoassay (EIA) for toxin

Assurance of Confidentiality: The voluntarily provided information obtained in this surveillance system that would permit identification of any individual or institution is collected with a guarantee that it will be held in strict confidence, will be used only for the purposes stated, and will not otherwise be disclosed or released without the consent of the individual, or the institution in accordance with Sections 304, 306 and 308(d) of the Public Health Service Act (42 USC 242b, 242k, and 242m(d)).
CDC 57.103 (Front) Rev. 15, v12.0

Public reporting burden of this collection of information is estimated to average 135 minutes per response, including the time for reviewing instructions, searching existing data sources, gathering, and maintaining the data needed, and completing and reviewing the collection of information. An agency may not conduct or sponsor, and a person is not required to respond to a collection of information unless it displays a currently valid OMB control number. Send comments regarding this burden estimate or any other aspect of this collection of information, including suggestions for reducing this burden to CDC, Reports Clearance Officer, 1600 Clifton Rd., MS H-21, Atlanta, GA 30333, ATTN: PRA (0920-0666).

- ☐ Cell cytotoxicity neutralization assay
- ☐ Nucleic acid amplification test (NAAT) (for example, PCR, LAMP)
- ☐ NAAT plus EIA, if NAAT positive (2-step algorithm)
- ☐ Glutamate dehydrogenase (GDH) antigen plus EIA for toxin (2-step algorithm)
- ☐ GDH plus NAAT (2-step algorithm)
- ☐ GDH plus EIA for toxin, followed by NAAT for discrepant results
- ☐ Toxigenic culture (*C. difficile* culture followed by detection of toxins)
- ☐ Other (specify): ______________________________

*22. Indicate the primary and definitive method used to identify microbes from blood cultures collected in your facility. (check one)

- ☐ MALDI-TOF MS System (Vitek MS)
- ☐ MALDI-TOF MS System (Bruker Biotyper)
- ☐ Automated Instrument (for example, Vitek, MicroScan, Phoenix, OmniLog, Sherlock, etc.)
- ☐ Non-automated Manual Kit (for example, API, Crystal, RapID, etc.)
- ☐ Rapid Identification (for example, Verigene, BioFire FilmArray, PNA-FISH, Gene Xpert, etc.)
- ☐ 16S rRNA Sequencing
- ☐ Other (specify): ________________
- ☐ None

*23. Indicate any additional secondary methods used for microbe identification from blood cultures collected in your facility (for example, a rapid method that is confirmed with the primary method, a secondary method if the primary method fails to give an identification, or a method that is used in conjunction with the primary method). (check all that apply)

- ☐ MALDI-TOF MS System (Vitek MS)
- ☐ MALDI-TOF MS System (Bruker Biotyper)
- ☐ Automated Instrument (for example, Vitek, MicroScan, Phoenix, OmniLog, Sherlock, etc.)

Facility Microbiology Laboratory Practices (continued)

- ☐ Non-automated Manual Kit (for example, API, Crystal, RapID, etc.)
- ☐ Rapid Identification (for example, Verigene, BioFire FilmArray, PNA-FISH, Gene Xpert, etc.)
- ☐ 16S rRNA Sequencing
- ☐ Other (specify): ________________
- ☐ None

Infection Control Practices
(completed with input from Hospital Epidemiologist and/or Quality Improvement Coordinator)

*24. Number or fraction of infection preventionists (IPs) in facility:

a. Total hours per week performing surveillance: __________________

Assurance of Confidentiality: The voluntarily provided information obtained in this surveillance system that would permit identification of any individual or institution is collected with a guarantee that it will be held in strict confidence, will be used only for the purposes stated, and will not otherwise be disclosed or released without the consent of the individual, or the institution in accordance with Sections 304, 306 and 308(d) of the Public Health Service Act (42 USC 242b, 242k, and 242m(d)).

Public reporting burden of this collection of information is estimated to average 135 minutes per response, including the time for reviewing instructions, searching existing data sources, gathering, and maintaining the data needed, and completing and reviewing the collection of information. An agency may not conduct or sponsor, and a person is not required to respond to a collection of information unless it displays a currently valid OMB control number. Send comments regarding this burden estimate or any other aspect of this collection of information, including suggestions for reducing this burden to CDC, Reports Clearance Officer, 1600 Clifton Rd., MS H-21, Atlanta, GA 30333, ATTN: PRA (0920-0666).

b. Total hours per week for infection control activities other than surveillance: ____________________

*25. Number or fraction of full-time employees (FTEs) for a designated hospital epidemiologist (or equivalent role) affiliated with your facility: ____________________

*26. Is it a policy in your facility that patients infected or colonized with MRSA are routinely placed in contact precautions while these patients are in your facility? (check one)

- ☐ Yes
- ☐ No
- ☐ Not applicable: my facility never admits these patients

26a. If Yes, check the type of patients that are routinely placed in contact precautions while in your facility (check one):

- ☐ All infected and all colonized patients
- ☐ Only all infected patients
- ☐ Only infected or colonized patients with certain characteristics (check all that apply)
  - ☐ Patients admitted to high risk settings
  - ☐ Patients at high risk for transmission

*27. Is it a policy in your facility that patients infected or colonized with VRE are routinely placed in contact precautions while these patients are in your facility? (check one)

- ☐ Yes
- ☐ No
- ☐ Not applicable: my facility never admits these patients

27a. If Yes, check the type of patients that are routinely placed in contact precautions while in your facility (check one):

- ☐ All infected and all colonized patients
- ☐ Only all infected patients
- ☐ Only infected or colonized patients with certain characteristics (check all that apply)
  - ☐ Patients admitted to high risk settings
  - ☐ Patients at high risk for transmission

Infection Control Practices (continued)

*28. Is it a policy in your facility that patients infected or colonized with CRE (regardless of confirmatory testing for carbapenemase production) are routinely placed in contact precautions while these patients are in your facility? (check one)

- ☐ Yes
- ☐ No

Assurance of Confidentiality: The voluntarily provided information obtained in this surveillance system that would permit identification of any individual or institution is collected with a guarantee that it will be held in strict confidence, will be used only for the purposes stated, and will not otherwise be disclosed or released without the consent of the individual, or the institution in accordance with Sections 304, 306 and 308(d) of the Public Health Service Act (42 USC 242b, 242k, and 242m(d)).
CDC 57.103 (Front) Rev. 15, v12.0

Public reporting burden of this collection of information is estimated to average 135 minutes per response, including the time for reviewing instructions, searching existing data sources, gathering, and maintaining the data needed, and completing and reviewing the collection of information. An agency may not conduct or sponsor, and a person is not required to respond to a collection of information unless it displays a currently valid OMB control number. Send comments regarding this burden estimate or any other aspect of this collection of information, including suggestions for reducing this burden to CDC, Reports Clearance Officer, 1600 Clifton Rd., MS H-21, Atlanta, GA 30333, ATTN: PRA (0920-0666).

☐ Not applicable: my facility never admits these patients

28a. If Yes, check the type of patients that are routinely placed in contact precautions while in your facility (check one):

☐ All infected and all colonized patients
☐ Only all infected patients
☐ Only infected or colonized patients with certain characteristics (check all that apply)
  ☐ Patients admitted to high risk settings
  ☐ Patients at high risk for transmission

*29. Is it a policy in your facility that patients infected or colonized with suspected or confirmed ESBL-producing or extended spectrum cephalosporin resistant *Enterobacterales* are routinely placed in contact precautions while these patients are in your facility? (check one)

☐ Yes
☐ No
☐ Not applicable: my facility never admits these patients

29a. If Yes, check the type of patients that are routinely placed in contact precautions while in your facility (check one):

☐ All infected and all colonized patients
☐ Only all infected patients
☐ Only infected or colonized patients with certain characteristics (check all that apply)
  ☐ Patients admitted to high risk settings
  ☐ Patients at high risk for transmission

*30. Does the facility routinely perform screening testing (culture or non-culture) for CRE? *This includes screening for patients at your facility performed by public health laboratories and commercial laboratories.*

☐ Yes ☐ No

30a. If Yes, in which situations does the facility routinely perform screening testing for CRE? (check all that apply)

☐ Surveillance testing at admission for all patients
☐ Surveillance testing of epidemiologically-linked patients of newly identified CRE patients (for example, roommates)
☐ Surveillance testing at admission of high-risk patients (check all that apply)
  ☐ Patients admitted from long-term acute care (LTAC) or long-term care facility (LTCF)
  ☐ Patients with recent (for example, within 6 months) overnight hospital stay outside the United States
  ☐ Patients admitted to high-risk settings (for example, ICU)
  ☐ Other high-risk patients (specify): _________________

Assurance of Confidentiality: The voluntarily provided information obtained in this surveillance system that would permit identification of any individual or institution is collected with a guarantee that it will be held in strict confidence, will be used only for the purposes stated, and will not otherwise be disclosed or released without the consent of the individual, or the institution in accordance with Sections 304, 306 and 308(d) of the Public Health Service Act (42 USC 242b, 242k, and 242m(d)).
CDC 57.103 (Front) Rev. 15, v12.0

Public reporting burden of this collection of information is estimated to average 135 minutes per response, including the time for reviewing instructions, searching existing data sources, gathering, and maintaining the data needed, and completing and reviewing the collection of information. An agency may not conduct or sponsor, and a person is not required to respond to a collection of information unless it displays a currently valid OMB control number. Send comments regarding this burden estimate or any other aspect of this collection of information, including suggestions for reducing this burden to CDC, Reports Clearance Officer, 1600 Clifton Rd., MS H-21, Atlanta, GA 30333, ATTN: PRA (0920-0666).

Infection Control Practices (continued)

- ☐ Surveillance testing of all patients in the facility or in a specific high-risk settings (for example, ICU) at pre-specified intervals (for example, weekly point prevalence survey)
- ☐ Other (specify): _________________

30b. If Yes, what method is routinely used by the lab conducting CRE testing of screening swabs from your facility? (check all that apply)

- ☐ Culture-based methods
- ☐ PCR
- ☐ Other (specify): _________________

*31. Does the facility routinely perform screening testing (culture or non-culture) for *Candida auris*? This includes screening for patients at your facility performed by public health laboratories and commercial laboratories. ☐ Yes ☐ No

31a. If Yes, in which situations does the facility routinely perform screening testing for *Candida auris*? (check all that apply)

- ☐ Surveillance testing at admission for all patients
- ☐ Surveillance testing of epidemiologically-linked patients of newly identified *Candida auris* patients (for example, point prevalence surveys in response to a case, patients in the same room or unit as a case)
- ☐ Surveillance testing at admission of high-risk patients (check all that apply)
  - ☐ Patients admitted from long-term acute care (LTAC) or long-term care facility (LTCF)
  - ☐ Patients with recent (for example, within 6 months) overnight hospital stay outside the United States
  - ☐ Patients admitted to high-risk settings (for example, ICU)
  - ☐ Other high-risk patients (specify): _________________
- ☐ Surveillance testing of all patients in the facility or in a specific high-risk setting (for example, ICU) at pre-specified intervals (for example, weekly point prevalence survey)
- ☐ Other (specify): _________________

31b. If Yes, what method is routinely used by the lab conducting *Candida auris* testing of screening swabs from your facility?

- ☐ Culture-based methods
- ☐ PCR
- ☐ Other (specify): _________________

*32. Does the facility routinely perform screening testing (culture or non-culture) for MRSA for any patients admitted to non-NICU settings? ☐ Yes ☐ No

32a. Does the facility routinely perform screening testing (culture or non-culture) for MRSA for any patients admitted to NICU settings?

Assurance of Confidentiality: The voluntarily provided information obtained in this surveillance system that would permit identification of any individual or institution is collected with a guarantee that it will be held in strict confidence, will be used only for the purposes stated, and will not otherwise be disclosed or released without the consent of the individual, or the institution in accordance with Sections 304, 306 and 308(d) of the Public Health Service Act (42 USC 242b, 242k, and 242m(d)).

Public reporting burden of this collection of information is estimated to average 135 minutes per response, including the time for reviewing instructions, searching existing data sources, gathering, and maintaining the data needed, and completing and reviewing the collection of information. An agency may not conduct or sponsor, and a person is not required to respond to a collection of information unless it displays a currently valid OMB control number. Send comments regarding this burden estimate or any other aspect of this collection of information, including suggestions for reducing this burden to CDC, Reports Clearance Officer, 1600 Clifton Rd., MS H-21, Atlanta, GA 30333, ATTN: PRA (0920-0666).

- ☐ Surveillance testing at admission for all patients
- ☐ Surveillance testing at admission of high-risk patients (for example, admitted from long-term acute care [LTAC] or long-term care facility [LTCF], or dialysis patients)
- ☐ Surveillance testing at admission of patients admitted to high-risk settings (for example, ICU)
- ☐ Surveillance testing of pre-operative patients to prevent surgical site infections
- ☐ Other (specify): _________________

Infection Control Practices (continued)

*33. Does the facility routinely perform screening testing (culture or non-culture) for MRSA for any patients admitted to NICU settings? ☐ Yes ☐ No

33a. If yes, in which situations does the facility routinely perform screening testing for MRSA for NICU settings? (check all that apply)

- ☐ Surveillance testing at admission for all patients
- ☐ Surveillance testing at admission for all transferred patients
- ☐ Surveillance testing of patients from known MRSA positive mothers
- ☐ Surveillance testing of high-risk patients (for example, infants born premature)
- ☐ Routine active surveillance testing (specifically, point prevalence surveys)
- ☐ Other (specify): _________________

*34. Does your facility have a policy to routinely use chlorhexidine bathing for any adult patients to prevent infection or transmission of MDROs at your facility?

☐ Yes ☐ No ☐ N/A, Children's Hospital

34a. If yes, indicate which patients: (select all that apply)

- ☐ ICU patients:
  - ○ All ICU patients
  - ○ Subset of ICU patients
    - ☐ Patients with central venous catheter or midline catheters
    - ☐ Others, specify:__________
- ☐ Patients outside the ICU:
  - ○ All patients outside the ICU
  - ○ Subset of patients outside the ICU
    - ☐ Patients with central venous catheter or midline catheters
    - ☐ Others, specify:__________
- ☐ Pre-operatively for patients undergoing surgery

*35. Does the facility have a policy to routinely use a combination of topical chlorhexidine <u>AND</u> an intranasal anti-staphylococcal agent (mupirocin, iodophor, or an alcohol based intranasal agent) for any adult patients to prevent healthcare-associated infections or reduce transmission of resistant pathogens?

☐ Yes ☐ No ☐ N/A, Children's Hospital

35a. If yes, indicate which patients: (select all that apply)

- ☐ ICU patients:
- ☐ Patients outside the ICU:

Assurance of Confidentiality: The voluntarily provided information obtained in this surveillance system that would permit identification of any individual or institution is collected with a guarantee that it will be held in strict confidence, will be used only for the purposes stated, and will not otherwise be disclosed or released without the consent of the individual, or the institution in accordance with Sections 304, 306 and 308(d) of the Public Health Service Act (42 USC 242b, 242k, and 242m(d)).
CDC 57.103 (Front) Rev. 15, v12.0

Public reporting burden of this collection of information is estimated to average 135 minutes per response, including the time for reviewing instructions, searching existing data sources, gathering, and maintaining the data needed, and completing and reviewing the collection of information. An agency may not conduct or sponsor, and a person is not required to respond to a collection of information unless it displays a currently valid OMB control number. Send comments regarding this burden estimate or any other aspect of this collection of information, including suggestions for reducing this burden to CDC, Reports Clearance Officer, 1600 Clifton Rd., MS H-21, Atlanta, GA 30333, ATTN: PRA (0920-0666).

☐ All ICU patients

☐ ICU patients who are known to be colonized or infected with MRSA

☐ ICU patients with central venous catheters or midline catheters

☐ Patients who are known to be colonized or infected with MRSA

☐ Patients with central venous catheters or midline catheters

☐ Pre-operatively for patients undergoing surgery

Assurance of Confidentiality: The voluntarily provided information obtained in this surveillance system that would permit identification of any individual or institution is collected with a guarantee that it will be held in strict confidence, will be used only for the purposes stated, and will not otherwise be disclosed or released without the consent of the individual, or the institution in accordance with Sections 304, 306 and 308(d) of the Public Health Service Act (42 USC 242b, 242k, and 242m(d)).

Public reporting burden of this collection of information is estimated to average 135 minutes per response, including the time for reviewing instructions, searching existing data sources, gathering, and maintaining the data needed, and completing and reviewing the collection of information. An agency may not conduct or sponsor, and a person is not required to respond to a collection of information unless it displays a currently valid OMB control number. Send comments regarding this burden estimate or any other aspect of this collection of information, including suggestions for reducing this burden to CDC, Reports Clearance Officer, 1600 Clifton Rd., MS H-21, Atlanta, GA 30333, ATTN: PRA (0920-0666).

Facility Neonatal or Newborn Patient Care Practices and Admissions Information

*36. Was this section completed in collaboration with your facility's neonatal or newborn patient care team? For example, was input sought from a neonatal or newborn patient care team member, such as a NICU Medical Director, Lead Neonatal Physician, Neonatal Nurse Manager, Lead Neonatal Nurse Practitioner?

- ☐ Yes
- ☐ No
- ☐ N/A, my facility does not provide neonatal or newborn patient care services at any level (specifically, my facility does **not** provide delivery services, Level 1 well newborn care, Level II special care, or neonatal intensive care)

If N/A was selected in question 36 above, questions 37-41 below do not apply to your facility and should be skipped. If your facility does care for neonates or newborns (at any level), complete questions below. *Questions should be answered based on the policies and practices that were in place for the majority of the last full calendar year.*

*37. Excluding Level I units (well newborn nurseries), record the number of neonatal admissions to Special Care Nurseries (Level II) and Intensive Care Units (Level II/III, Level III, Level IV):

a. Inborn Admissions: ______________________
b. Outborn Admissions: ______________________

*38. Excluding Level I units (well newborn nurseries), record the number of neonatal admissions (both inborn and outborn) to Special Care (Level II) and Intensive Care (Level II/III, Level III, Level IV) in each of following birth weight categories:

a. Less than or equal to 750 grams: _________________
b. 751-1000 grams: _________________
c. 1001-1500 grams: _____________________________
d. 1501-2500 grams: _________________________
e. More than 2500 grams: _____________________

*39. Does your facility provide Level III (or higher) neonatal intensive care as defined by the American Academy of Pediatrics (for example, capable of providing sustained life support, comprehensive care for infants born <32 weeks gestation and weighing <1500 grams, a full range of respiratory support that may include conventional and/or high-frequency ventilation)?

*40. Does your facility accept neonates as transfers for any of the following procedures: Omphalocele repair; ventriculoperitoneal shunt; tracheoesophageal fistula (TEF)/esophageal atresia repair; bowel resection/reanastomosis; meningomyelocele repair; cardiac catheterization?

☐ Yes ☐ No

<u>To help us better understand your facility's practices and protocols for administering antimicrobials to newborns, answer the following questions:</u>

*41. If babies are roomed with their mother in a labor and delivery or postpartum ward and are administered oral or parenteral antimicrobials, such as ampicillin, what location is the medication administration attributed to in the

Assurance of Confidentiality: The voluntarily provided information obtained in this surveillance system that would permit identification of any individual or institution is collected with a guarantee that it will be held in strict confidence, will be used only for the purposes stated, and will not otherwise be disclosed or released without the consent of the individual, or the institution in accordance with Sections 304, 306 and 308(d) of the Public Health Service Act (42 USC 242b, 242k, and 242m(d)).
CDC 57.103 (Front) Rev. 15, v12.0

Public reporting burden of this collection of information is estimated to average 135 minutes per response, including the time for reviewing instructions, searching existing data sources, gathering, and maintaining the data needed, and completing and reviewing the collection of information. An agency may not conduct or sponsor, and a person is not required to respond to a collection of information unless it displays a currently valid OMB control number. Send comments regarding this burden estimate or any other aspect of this collection of information, including suggestions for reducing this burden to CDC, Reports Clearance Officer, 1600 Clifton Rd., MS H-21, Atlanta, GA 30333, ATTN: PRA (0920-0666).

electronic medication administration record (eMAR) system and/or bar code medication administration (BCMA) system?

- ☐ a. Level I Well Newborn Nursery
- ☐ b. Labor and Delivery Ward, Postpartum Ward, or Labor, Delivery, Recovery, Postpartum Suite
- ☐ c. My facility requires that babies receiving antimicrobials **intravenously** (IV) are transferred out of their mother's room in order for IV antimicrobials to be administered (babies receiving oral or intramuscular antimicrobials may remain in their mother's room for antimicrobial administration)

Neonatal or Newborn Patient Care Practices and Admissions (continued)

- ☐ d. My facility requires that babies receiving oral **and/or** intramuscular antimicrobials are transferred out of their mother's room in order for antimicrobials to be administered
- ☐ e. N/A my facility does not provide delivery services

41a. If answer choice **c.** or **d.** was selected above, to which neonatal unit would a baby be transferred in order to receive oral or parenteral antimicrobials (select all that apply):

- ☐ Level I Well Newborn Nursery separate from the mother's room
- ☐ Level II Special Care Nursery
- ☐ Level II/III or higher Neonatal Intensive Care Unit

Antibiotic Stewardship Practices (completed with input from Physician and Pharmacist Stewardship Leaders)

*42. Did the antibiotic stewardship leader(s) participate in responding to these questions? (Check one.)

- ☐ Yes, pharmacist lead
- ☐ Yes, physician lead
- ☐ Yes, both pharmacist and physician leads
- ☐ Yes, other lead
- ☐ No

*43. Facility leadership has demonstrated commitment to antibiotic stewardship efforts by: (Check all that apply.)

- ☐ Providing stewardship program leader(s) dedicated time to manage the program and conduct daily stewardship interventions.
- ☐ Allocating resources (for example, IT support, training for stewardship team) to support antibiotic stewardship efforts.
- ☐ Having a senior executive that serves as a point of contact or "champion" to help ensure the program has resources and support to accomplish its mission.
- ☐ Presenting information on stewardship activities and outcomes to facility leadership and/or board at least annually.

Assurance of Confidentiality: The voluntarily provided information obtained in this surveillance system that would permit identification of any individual or institution is collected with a guarantee that it will be held in strict confidence, will be used only for the purposes stated, and will not otherwise be disclosed or released without the consent of the individual, or the institution in accordance with Sections 304, 306 and 308(d) of the Public Health Service Act (42 USC 242b, 242k, and 242m(d)).
CDC 57.103 (Front) Rev. 15, v12.0

Public reporting burden of this collection of information is estimated to average 135 minutes per response, including the time for reviewing instructions, searching existing data sources, gathering, and maintaining the data needed, and completing and reviewing the collection of information. An agency may not conduct or sponsor, and a person is not required to respond to a collection of information unless it displays a currently valid OMB control number. Send comments regarding this burden estimate or any other aspect of this collection of information, including suggestions for reducing this burden to CDC, Reports Clearance Officer, 1600 Clifton Rd., MS H-21, Atlanta, GA 30333, ATTN: PRA (0920-0666).

- ☐ Ensuring the stewardship program has an opportunity to discuss resource needs with facility leadership and/or board at least annually.
- ☐ Communicating to staff about stewardship activities, via email, newsletters, events, or other avenues.
- ☐ Providing opportunities for hospital staff training and development on antibiotic stewardship.
- ☐ Providing a formal statement of support for antibiotic stewardship (for example, a written policy or statement approved by the board).
- ☐ Ensuring that staff from key support departments and groups (for example, IT and hospital medicine) are contributing to stewardship activities.
- ☐ None of the above

*44. Our facility has a leader or co-leaders responsible for antibiotic stewardship program management and outcomes. ☐ Yes ☐ No

44a. If Yes, what is the position of this leader? (Check one.)

- ☐ Physician
- ☐ Pharmacist

Antibiotic Stewardship Practices (continued)

- ☐ Co-led by both Pharmacist and Physician
- ☐ Other (for example, RN, PA, NP, etc.; specify):________________

44b. If Physician or Co-led is selected, which of the following describes your antibiotic stewardship **physician** leader? (Check all that apply.)

- ☐ Has antibiotic stewardship responsibilities in their contract job description, or performance review
- ☐ Is physically on-site in your facility (either part-time or full-time
- ☐ Completed an ID fellowship
- ☐ Completed a certificate program on antibiotic stewardship
- ☐ Completed other training(s) (for example, conferences or online modules) on antibiotic stewardship
- ☐ None of the above

44c. If 'Has antibiotic stewardship responsibilities in their contract or job description' is selected (for physician (co) leader): What percentage of time for antibiotic stewardship activities is specified in the **physician** (co) leader's **contract or job description**? (Check one.)

- ☐ 1-10%
- ☐ 11-25%
- ☐ 26-50%
- ☐ 51-75%
- ☐ 76-100%
- ☐ Not specified

44d. If Physician or Co-led is selected: **In an average week**, what percentage of time does the **physician** (co) leader **spend** on antibiotic stewardship activities in your facility? (Check one.)

- ☐ 1-10%
- ☐ 11-25%
- ☐ 26-50%
- ☐ 51-75%
- ☐ 76-100%

Assurance of Confidentiality: The voluntarily provided information obtained in this surveillance system that would permit identification of any individual or institution is collected with a guarantee that it will be held in strict confidence, will be used only for the purposes stated, and will not otherwise be disclosed or released without the consent of the individual, or the institution in accordance with Sections 304, 306 and 308(d) of the Public Health Service Act (42 USC 242b, 242k, and 242m(d)).
CDC 57.103 (Front) Rev. 15, v12.0

Public reporting burden of this collection of information is estimated to average 135 minutes per response, including the time for reviewing instructions, searching existing data sources, gathering, and maintaining the data needed, and completing and reviewing the collection of information. An agency may not conduct or sponsor, and a person is not required to respond to a collection of information unless it displays a currently valid OMB control number. Send comments regarding this burden estimate or any other aspect of this collection of information, including suggestions for reducing this burden to CDC, Reports Clearance Officer, 1600 Clifton Rd., MS H-21, Atlanta, GA 30333, ATTN: PRA (0920-0666).

44e. If Pharmacist or Co-led is selected, which of the following describes your antibiotic stewardship **pharmacist** leader? (Check all that apply.)

- ☐ Has antibiotic stewardship responsibilities in their contract, job description, or performance review
- ☐ Is physically on-site in your facility (either part-time or full-time)
- ☐ Completed a PGY2 ID residency and/or ID fellowship
- ☐ Completed a certificate program on antibiotic stewardship
- ☐ Completed other training(s) (for example, conferences or online modules) on antibiotic stewardship
- ☐ None of the above

44f. If 'Has antibiotic stewardship responsibilities in their contract or job description' is selected (for pharmacist (co) leader): What percent time for antibiotic stewardship activities is specified in the **pharmacist** (co) leader's **contract or job description**? (Check one)

- ☐ 1-10% ☐ 51-75%
- ☐ 11-25% ☐ 76-100%
- ☐ 26-50% ☐ Not specified

44g. If 'Pharmacist' or 'Co-led' is selected: **In an average week**, what percentage of time does the **pharmacist** (co) leader **spend** on antibiotic stewardship activities in your facility? (Check one)

- ☐ 1-10% ☐ 26-50% ☐ 76-100%
- ☐ 11-25% ☐ 51-75%

Antibiotic Stewardship Practices (continued)

44h. If Pharmacist or Other is selected: Does your facility have a designated physician who can serve as a point of contact and support for the non-physician leader?

☐ Yes ☐ No

44i. If a pharmacist is **not** the leader or co-leader for the program, is there at least one pharmacist responsible for improving antibiotic use at your facility?

☐ Yes ☐ No

*45. Our facility has the following priority antibiotic stewardship interventions: (Check all that apply)

- ☐ Prospective audit and feedback for specific antibiotic agents

45a. If Prospective audit and feedback is selected: For which categories of antimicrobials? Answer for the following categories of antimicrobials, *whether or not* they are on formulary. (Check all that apply)

- ☐ Cefepime, ceftazidime, or piperacillin/tazobactam
- ☐ Vancomycin (intravenous)
- ☐ Ertapenem, imipenem/cilastatin, or meropenem
- ☐ Ceftazidime/avibactam, ceftolozane/tazobactam, meropenem/vaborbactam, imipenem-cilastatin/relebactam, or cefiderocol

Assurance of Confidentiality: The voluntarily provided information obtained in this surveillance system that would permit identification of any individual or institution is collected with a guarantee that it will be held in strict confidence, will be used only for the purposes stated, and will not otherwise be disclosed or released without the consent of the individual, or the institution in accordance with Sections 304, 306 and 308(d) of the Public Health Service Act (42 USC 242b, 242k, and 242m(d)).

Public reporting burden of this collection of information is estimated to average 135 minutes per response, including the time for reviewing instructions, searching existing data sources, gathering, and maintaining the data needed, and completing and reviewing the collection of information. An agency may not conduct or sponsor, and a person is not required to respond to a collection of information unless it displays a currently valid OMB control number. Send comments regarding this burden estimate or any other aspect of this collection of information, including suggestions for reducing this burden to CDC, Reports Clearance Officer, 1600 Clifton Rd., MS H-21, Atlanta, GA 30333, ATTN: PRA (0920-0666).

- ☐ Fluoroquinolones
- ☐ Daptomycin, linezolid, or other newer anti-MRSA agents
- ☐ Eravacycline or omadacycline
- ☐ Lefamulin
- ☐ Aminoglycosides
- ☐ Colistin or polymyxin B
- ☐ Anidulafungin, caspofungin, or micafungin
- ☐ Isavuconazole, posaconazole, or voriconazole
- ☐ Amphotericin B and/or lipid-based amphotericin B
- ☐ None of the above

45b. If Prospective audit and feedback is selected: Our antibiotic stewardship program monitors prospective audit and feedback interventions (for example, by tracking antibiotic use, types of interventions, acceptance of recommendations).

☐ Yes ☐ No

☐ Preauthorization for specific antibiotic agents.

45c. If Preauthorization is selected: For which categories of antimicrobials? Only answer for categories of antimicrobials that are ***on formulary***. (Check all that apply)

- ☐ Cefepime, ceftazidime, or piperacillin/tazobactam
- ☐ Vancomycin (intravenous)
- ☐ Ertapenem, imipenem/cilastatin, or meropenem
- ☐ Ceftazidime/avibactam, ceftolozane/tazobactam, meropenem/vaborbactam, imipenem-cilastatin/relebactam, or cefiderocol
- ☐ Fluoroquinolones
- ☐ Daptomycin, linezolid, or other newer anti-MRSA agents
- ☐ Eravacycline or omadacycline

Antibiotic Stewardship Practices (continued)

- ☐ Lefamulin
- ☐ Aminoglycosides
- ☐ Colistin or polymyxin B
- ☐ Anidulafungin, caspofungin, or micafungin
- ☐ Isavuconazole, posaconazole, or voriconazole
- ☐ Amphotericin B and/or lipid-based amphotericin B
- ☐ None of the above

45d. If Preauthorization is selected: Our antibiotic stewardship program monitors preauthorization interventions (for example, by tracking which agents are requested for which conditions).

Assurance of Confidentiality: The voluntarily provided information obtained in this surveillance system that would permit identification of any individual or institution is collected with a guarantee that it will be held in strict confidence, will be used only for the purposes stated, and will not otherwise be disclosed or released without the consent of the individual, or the institution in accordance with Sections 304, 306 and 308(d) of the Public Health Service Act (42 USC 242b, 242k, and 242m(d)).

Public reporting burden of this collection of information is estimated to average 135 minutes per response, including the time for reviewing instructions, searching existing data sources, gathering, and maintaining the data needed, and completing and reviewing the collection of information. An agency may not conduct or sponsor, and a person is not required to respond to a collection of information unless it displays a currently valid OMB control number. Send comments regarding this burden estimate or any other aspect of this collection of information, including suggestions for reducing this burden to CDC, Reports Clearance Officer, 1600 Clifton Rd., MS H-21, Atlanta, GA 30333, ATTN: PRA (0920-0666).

☐ Yes ☐ No

☐ Facility-specific treatment recommendations, based on national guidelines and local pathogen susceptibilities, to assist with antibiotic selection for common clinical conditions (for example, community-acquired pneumonia, urinary tract infection, skin and soft tissue infection)

45e. If Facility-specific treatment recommendations is selected: For which common clinical conditions?
- ☐ Community-acquired pneumonia
- ☐ Urinary tract infection
- ☐ Skin and soft tissue infection
- ☐ None of the above

45f. If Facility-specific treatment recommendations is selected: Our stewardship program monitors adherence to our facility's treatment recommendations for antibiotic selection for common clinical conditions (for example, community-acquired pneumonia, urinary tract infection, skin and soft tissue infection).

☐ Yes ☐ No

45g. If Yes: For which common clinical conditions?
- ☐ Community-acquired pneumonia
- ☐ Urinary tract infection
- ☐ Skin and soft tissue infection
- ☐ None of the above

☐ None of the above

*46. Our facility has a policy or formal procedure for other interventions to ensure optimal use of antibiotics: (Check all that apply.)
- ☐ Early administration of effective antibiotics to optimize the treatment of sepsis
- ☐ Treatment protocols for *Staphylococcus aureus* bloodstream infection
- ☐ Stopping unnecessary antibiotic(s) in new cases of *Clostridioides difficile* infection (CDI)
- ☐ Review of culture-proven invasive (for example, bloodstream) infections
- ☐ Review of planned outpatient parenteral antibiotic therapy (OPAT)
- ☐ The treating team to review antibiotics 48-72 hours after initial order (specifically, antibiotic time-out).
- ☐ Assess and clarify documented penicillin allergy

Antibiotic Stewardship Practices (continued)

- ☐ Using the shortest effective duration of antibiotics at discharge for common clinical conditions (for example, community-acquired pneumonia, urinary tract infections, skin, and soft tissue infections)
- ☐ None of the above

Assurance of Confidentiality: The voluntarily provided information obtained in this surveillance system that would permit identification of any individual or institution is collected with a guarantee that it will be held in strict confidence, will be used only for the purposes stated, and will not otherwise be disclosed or released without the consent of the individual, or the institution in accordance with Sections 304, 306 and 308(d) of the Public Health Service Act (42 USC 242b, 242k, and 242m(d)).

Public reporting burden of this collection of information is estimated to average 135 minutes per response, including the time for reviewing instructions, searching existing data sources, gathering, and maintaining the data needed, and completing and reviewing the collection of information. An agency may not conduct or sponsor, and a person is not required to respond to a collection of information unless it displays a currently valid OMB control number. Send comments regarding this burden estimate or any other aspect of this collection of information, including suggestions for reducing this burden to CDC, Reports Clearance Officer, 1600 Clifton Rd., MS H-21, Atlanta, GA 30333, ATTN: PRA (0920-0666).

46a. If ‘Using the shortest effective duration of antibiotics at discharge for common clinical conditions’ is selected: Our stewardship program monitors adherence in using the shortest effective duration of antibiotics at discharge for common clinical conditions (for example, community-acquired pneumonia, urinary tract infections, skin and soft tissue infections), at least annually.

☐ Yes ☐ No

*47. Our facility has in place the following specific ‘pharmacy-based’ interventions: (Check all that apply)

- ☐ Pharmacy-driven changes from intravenous to oral antibiotics without a physician’s order (for example, hospital-approved protocol)
- ☐ Alerts to providers about potentially duplicative antibiotic spectra (for example, multiple antibiotics to treat anaerobes)
- ☐ Automatic antibiotic stop orders in specific situations (for example, surgical prophylaxis)
- ☐ None of the above

*48. Our stewardship program has engaged bedside nurses in actions to optimize antibiotic use.

☐ Yes ☐ No

48a. If Yes is selected: Our facility has in place the following specific ‘nursing-based’ interventions: (Check all that apply.)

- ☐ Nurses receive training on appropriate criteria for sending urine and/or respiratory cultures.
- ☐ Nurses initiate discussions with the treating team on switching from intravenous to oral antibiotics.
- ☐ Nurses initiate antibiotic time-out discussions with the treating team.
- ☐ Nurses track antibiotic duration of therapy.
- ☐ None of the above

48b. If ‘Nurses track antibiotic duration of therapy’ is selected: Is that information available at the bedside (for example, on a whiteboard in the room)?

☐ Yes ☐ No

*49. Our stewardship program monitors: (Check all that apply.)

- ☐ Antibiotic resistance patterns (either facility- or region-specific), at least annually
- ☐ *Clostridioides difficile* infections (or *C. difficile* LabID events), at least annually
- ☐ Antibiotic use in days of therapy (DOT) per 1000 patient days or days present, at least quarterly
- ☐ Antibiotic use in defined daily doses (DDD) per 1000 patient days, at least quarterly
- ☐ Antibiotic expenditures (specifically, purchasing costs), at least quarterly
- ☐ Antibiotic use in some other way, at least annually (specify): ___________________
- ☐ None of the above

Assurance of Confidentiality: The voluntarily provided information obtained in this surveillance system that would permit identification of any individual or institution is collected with a guarantee that it will be held in strict confidence, will be used only for the purposes stated, and will not otherwise be disclosed or released without the consent of the individual, or the institution in accordance with Sections 304, 306 and 308(d) of the Public Health Service Act (42 USC 242b, 242k, and 242m(d)).
CDC 57.103 (Front) Rev. 15, v12.0

Public reporting burden of this collection of information is estimated to average 135 minutes per response, including the time for reviewing instructions, searching existing data sources, gathering, and maintaining the data needed, and completing and reviewing the collection of information. An agency may not conduct or sponsor, and a person is not required to respond to a collection of information unless it displays a currently valid OMB control number. Send comments regarding this burden estimate or any other aspect of this collection of information, including suggestions for reducing this burden to CDC, Reports Clearance Officer, 1600 Clifton Rd., MS H-21, Atlanta, GA 30333, ATTN: PRA (0920-0666).

Antibiotic Stewardship Practices (continued)

*50. Our stewardship team provides the following antibiotic use reports to prescribers, at least annually: (Check all that apply.)

☐ Individual, prescriber-level reports

☐ Unit- or service-specific reports

☐ None of the above

50a. If 'Individual, prescriber-level reports' or 'Unit- or service-specific reports' is selected: Our stewardship program uses these reports to target feedback to prescribers about how they can improve their antibiotic prescribing, at least annually.

☐ Yes ☐ No

*51. Our facility distributes an antibiogram to prescribers, at least annually.

☐ Yes ☐ No

*52. Information on antibiotic use, antibiotic resistance, and stewardship efforts is reported to hospital staff, at least annually.

☐ Yes ☐ No

*53. Which of the following groups receive education on optimal prescribing, adverse reactions from antibiotics, and antibiotic resistance (for example, Grand Rounds, in-service training, direct instruction) at least annually? (Check all that apply.)

☐ Prescribers

☐ Nursing staff

☐ Pharmacists

☐ None of the above

*54. Are patients provided education on important side effects of prescribed antibiotics?

☐ Yes ☐ No

54a. If 'Yes' is selected: How is education to patients on side effects shared? (Check all that apply.)

☐ Discharge paperwork

☐ Verbally by nurse

☐ Verbally by pharmacist

☐ Verbally by physician

☐ None of the above

Optional Antibiotic Stewardship Practices Questions
Responses to the following questions are not required to complete the annual survey.
Provide additional information about your facility's antibiotic stewardship activities and leadership.

Assurance of Confidentiality: The voluntarily provided information obtained in this surveillance system that would permit identification of any individual or institution is collected with a guarantee that it will be held in strict confidence, will be used only for the purposes stated, and will not otherwise be disclosed or released without the consent of the individual, or the institution in accordance with Sections 304, 306 and 308(d) of the Public Health Service Act (42 USC 242b, 242k, and 242m(d)).

Public reporting burden of this collection of information is estimated to average 135 minutes per response, including the time for reviewing instructions, searching existing data sources, gathering, and maintaining the data needed, and completing and reviewing the collection of information. An agency may not conduct or sponsor, and a person is not required to respond to a collection of information unless it displays a currently valid OMB control number. Send comments regarding this burden estimate or any other aspect of this collection of information, including suggestions for reducing this burden to CDC, Reports Clearance Officer, 1600 Clifton Rd., MS H-21, Atlanta, GA 30333, ATTN: PRA (0920-0666).

55. Antibiotic stewardship activities are integrated into quality improvement and/or patient safety initiatives.

☐ Yes ☐ No

Assurance of Confidentiality: The voluntarily provided information obtained in this surveillance system that would permit identification of any individual or institution is collected with a guarantee that it will be held in strict confidence, will be used only for the purposes stated, and will not otherwise be disclosed or released without the consent of the individual, or the institution in accordance with Sections 304, 306 and 308(d) of the Public Health Service Act (42 USC 242b, 242k, and 242m(d)).

Public reporting burden of this collection of information is estimated to average 135 minutes per response, including the time for reviewing instructions, searching existing data sources, gathering, and maintaining the data needed, and completing and reviewing the collection of information. An agency may not conduct or sponsor, and a person is not required to respond to a collection of information unless it displays a currently valid OMB control number. Send comments regarding this burden estimate or any other aspect of this collection of information, including suggestions for reducing this burden to CDC, Reports Clearance Officer, 1600 Clifton Rd., MS H-21, Atlanta, GA 30333, ATTN: PRA (0920-0666).

Optional Antibiotic Stewardship Practices (continued)

56. Our facility accesses targeted remote stewardship expertise (for example, tele-stewardship to obtain facility-specific support for our antibiotic stewardship efforts).

☐ Yes ☐ No

57. Our stewardship program works with the microbiology laboratory to implement the following interventions: (Check all that apply)
    - ☐ Selective reporting of antimicrobial susceptibility testing results
    - ☐ Placing comments in microbiology reports to improve prescribing
    - ☐ None of the above

58. Which committees or leadership entities provide oversight of your facility's antibiotic stewardship efforts? (Check all that apply)
    - ☐ Pharmacy director
    - ☐ Pharmacy & therapeutics
    - ☐ Patient safety
    - ☐ Quality improvement
    - ☐ Executive leadership (for example, CEO, CMO)
    - ☐ Hospital board
    - ☐ Other (specify): _______________
    - ☐ None

Sepsis Management and Practices

*59. Our facility has a program or committee charged with monitoring and reviewing improving sepsis care and/or outcomes.

☐ Yes ☐ No

59a. If Yes: The responsibilities of this committee include the following: (Check all that apply; check at least one)
- ☐ Developing and updating hospital sepsis guidelines
- ☐ Developing and updating hospital sepsis order sets
- ☐ Monitor and review compliance with Centers for Medicare & Medicaid SEP-1 measure
- ☐ Monitor and review effectiveness of early sepsis identification strategies
- ☐ Monitoring and reviewing management of patients with sepsis
- ☐ Monitor and review outcomes among patients with sepsis
- ☐ Monitor and review antimicrobial use in sepsis in conjunction with antimicrobial stewardship or infectious disease staff
- ☐ Providing education to hospital staff on sepsis
- ☐ Setting annual goals for sepsis management and/or outcomes
- ☐ None of the above

Assurance of Confidentiality: The voluntarily provided information obtained in this surveillance system that would permit identification of any individual or institution is collected with a guarantee that it will be held in strict confidence, will be used only for the purposes stated, and will not otherwise be disclosed or released without the consent of the individual, or the institution in accordance with Sections 304, 306 and 308(d) of the Public Health Service Act (42 USC 242b, 242k, and 242m(d)).
CDC 57.103 (Front) Rev. 15, v12.0

Public reporting burden of this collection of information is estimated to average 135 minutes per response, including the time for reviewing instructions, searching existing data sources, gathering, and maintaining the data needed, and completing and reviewing the collection of information. An agency may not conduct or sponsor, and a person is not required to respond to a collection of information unless it displays a currently valid OMB control number. Send comments regarding this burden estimate or any other aspect of this collection of information, including suggestions for reducing this burden to CDC, Reports Clearance Officer, 1600 Clifton Rd., MS H-21, Atlanta, GA 30333, ATTN: PRA (0920-0666).

Assurance of Confidentiality: The voluntarily provided information obtained in this surveillance system that would permit identification of any individual or institution is collected with a guarantee that it will be held in strict confidence, will be used only for the purposes stated, and will not otherwise be disclosed or released without the consent of the individual, or the institution in accordance with Sections 304, 306 and 308(d) of the Public Health Service Act (42 USC 242b, 242k, and 242m(d)).
CDC 57.103 (Front) Rev. 15, v12.0

Public reporting burden of this collection of information is estimated to average 135 minutes per response, including the time for reviewing instructions, searching existing data sources, gathering, and maintaining the data needed, and completing and reviewing the collection of information. An agency may not conduct or sponsor, and a person is not required to respond to a collection of information unless it displays a currently valid OMB control number. Send comments regarding this burden estimate or any other aspect of this collection of information, including suggestions for reducing this burden to CDC, Reports Clearance Officer, 1600 Clifton Rd., MS H-21, Atlanta, GA 30333, ATTN: PRA (0920-0666).

Sepsis Management and Practices (continued)

59b. If Yes: This program or committee includes representatives with the following backgrounds healthcare personnel: (Check all that apply; check at least one)

- ☐ Physician
- ☐ Nurse
- ☐ Pharmacist
- ☐ Advanced practice provider (for example, Physician Assistant, Nurse Practitioner
- ☐ Social worker
- ☐ Quality improvement staff member
- ☐ Case manager
- ☐ Microbiology laboratory staff member
- ☐ Discharge planner
- ☐ None of the above

59c. If Yes:, This program or committee includes representatives from the following hospital locations or services (Check all that apply; check at least one)

- ☐ Antimicrobial Stewardship
- ☐ Critical Care / Intensive Care (excluding Neonatal Intensive Care)
- ☐ Data Analytics
- ☐ Emergency Medicine
- ☐ Hospital Medicine
- ☐ Infectious Diseases
- ☐ Information Technology
- ☐ Laboratory
- ☐ Neonatal Intensive Care
- ☐ Obstetrics/Labor and Deliver
- ☐ Pediatrics
- ☐ Pharmacy
- ☐ None of the above

*60. Our facility has one leader or two co-leaders responsible for sepsis program or committee management and outcomes. (Check one)

- ☐ Yes
- ☐ No (we have no designated leaders)
- ☐ No (we have more than 2 leaders)

60a. If yes selected in 60: What is the professional background of the sepsis program or committee leaders(s)?

- ☐ Advanced practice provider (APP)
- ☐ Nurse
- ☐ Physician
- ☐ None of the above

60b. If Yes selected in 60: Did the sepsis program leader(s) participate in responding to these questions? (Check one)

Assurance of Confidentiality: The voluntarily provided information obtained in this surveillance system that would permit identification of any individual or institution is collected with a guarantee that it will be held in strict confidence, will be used only for the purposes stated, and will not otherwise be disclosed or released without the consent of the individual, or the institution in accordance with Sections 304, 306 and 308(d) of the Public Health Service Act (42 USC 242b, 242k, and 242m(d)).

Public reporting burden of this collection of information is estimated to average 135 minutes per response, including the time for reviewing instructions, searching existing data sources, gathering, and maintaining the data needed, and completing and reviewing the collection of information. An agency may not conduct or sponsor, and a person is not required to respond to a collection of information unless it displays a currently valid OMB control number. Send comments regarding this burden estimate or any other aspect of this collection of information, including suggestions for reducing this burden to CDC, Reports Clearance Officer, 1600 Clifton Rd., MS H-21, Atlanta, GA 30333, ATTN: PRA (0920-0666).

- ☐ Yes
- ☐ No

Assurance of Confidentiality: The voluntarily provided information obtained in this surveillance system that would permit identification of any individual or institution is collected with a guarantee that it will be held in strict confidence, will be used only for the purposes stated, and will not otherwise be disclosed or released without the consent of the individual, or the institution in accordance with Sections 304, 306 and 308(d) of the Public Health Service Act (42 USC 242b, 242k, and 242m(d)).

Public reporting burden of this collection of information is estimated to average 135 minutes per response, including the time for reviewing instructions, searching existing data sources, gathering, and maintaining the data needed, and completing and reviewing the collection of information. An agency may not conduct or sponsor, and a person is not required to respond to a collection of information unless it displays a currently valid OMB control number. Send comments regarding this burden estimate or any other aspect of this collection of information, including suggestions for reducing this burden to CDC, Reports Clearance Officer, 1600 Clifton Rd., MS H-21, Atlanta, GA 30333, ATTN: PRA (0920-0666).

Sepsis Management and Practices (continued)

60c. If APP selected in #60a: What percentage of the APP leader's effort is specified for sepsis activities? If there are two APP leaders, please indicate the sum of their combined effort if it were applied towards a single APP. (Check one)

- ☐ 0% (Sepsis activities are voluntary with no specified effort)
- ☐ 1 to 10%
- ☐ 11 to 25%
- ☐ 26 to 50%
- ☐ More than 50%
- ☐ Not specified

60d. If nurse selected in #60a.: What percentage of the nurse leader's effort is specified for sepsis activities? If there are two nurse leaders, please indicate the sum of their combined effort if it were applied towards a single nurse. (Check one)

- ☐ 0% (Sepsis activities are voluntary with no specified effort)
- ☐ 1 to 10%
- ☐ 11 to 25%
- ☐ 26 to 50%
- ☐ More than 50%
- ☐ Not specified

60e. If physician selected in #60a.: What percentage of the physician leader's effort is specified for sepsis activities? If there are two physician leaders, please indicated the sum of their combined effort if it were applied towards a single physician.

- ☐ 0% (Sepsis activities are voluntary with no specified effort)
- ☐ 1 to 10%
- ☐ 11 to 25%
- ☐ 26 to 50%
- ☐ More than 50%
- ☐ Not specified

*61. Facility leadership has demonstrated commitment to improving sepsis care by: (Check all that apply; check at least one.)

- ☐ Providing sepsis program leader(s) with sufficient specified time to manage the hospital sepsis program.
- ☐ Providing sufficient resources, including data analytics and information technology support, to operate the program effectively.
- ☐ Ensuring that relevant staff from key clinical groups and support departments have sufficient time to contribute to sepsis activities.
- ☐ Appointing a senior leader to serve as an executive sponsor for the sepsis program.
- ☐ Identifying sepsis as a facility priority and communicating this priority to hospital staff.
- ☐ None of the above.

*62. Our facility uses the following approaches to assist in the rapid identification of patients with sepsis <u>upon presentation</u> to the facility: (Check all that apply; check at least one.)

Assurance of Confidentiality: The voluntarily provided information obtained in this surveillance system that would permit identification of any individual or institution is collected with a guarantee that it will be held in strict confidence, will be used only for the purposes stated, and will not otherwise be disclosed or released without the consent of the individual, or the institution in accordance with Sections 304, 306 and 308(d) of the Public Health Service Act (42 USC 242b, 242k, and 242m(d)).

Public reporting burden of this collection of information is estimated to average 135 minutes per response, including the time for reviewing instructions, searching existing data sources, gathering, and maintaining the data needed, and completing and reviewing the collection of information. An agency may not conduct or sponsor, and a person is not required to respond to a collection of information unless it displays a currently valid OMB control number. Send comments regarding this burden estimate or any other aspect of this collection of information, including suggestions for reducing this burden to CDC, Reports Clearance Officer, 1600 Clifton Rd., MS H-21, Atlanta, GA 30333, ATTN: PRA (0920-0666).

- ☐ Manual screening for clinical instability (e.g., MEWS, NEWS score)
- ☐ Electronic health record (EHR)-based screening for clinical instability
- ☐ Manual screening for sepsis criteria
- ☐ Electronic Health Record (HER)-based screening for sepsis criteria
- ☐ None of the above

Sepsis Management and Practices (continued)

*63. Our facility uses the following approaches to assist in identification of sepsis throughout hospitalization: (Check all that apply; check at least one.)

- ☐ Manual screening for clinical instability (e.g., MEWS, NEWS score)
- ☐ Electronic health record (EHR)-based screening for clinical instability
- ☐ Manual screening for sepsis criteria
- ☐ Electronic Health Record (EHR)-based screening for sepsis criteria
- ☐ None of the above

*64. Our facility uses the following approaches to promote evidence-based management of patients with sepsis: (Check all that apply; check at least one.)

- ☐ Hospital guideline or care pathway for management of sepsis
- ☐ Hospital order set for management of sepsis
- ☐ Structured template for documentation of sepsis treatment
- ☐ Standardized process for verbal hand-off of sepsis treatment
- ☐ Sepsis Response Team
- ☐ Rapid Response Team with training in sepsis management
- ☐ None of the above

*65. Our facility uses the following approaches to promote rapid antimicrobial delivery to patients with sepsis: (Check all that apply; check at least one.)

- ☐ Stocking of common antimicrobials in locations outside the pharmacy
- ☐ Immediate processing of new antimicrobial orders in patients with sepsis
- ☐ Orders that default to ordering immediate administration of new antimicrobials
- ☐ Pharmacists on-site in key locations outside the pharmacy
- ☐ None of the above

*66. Our facility uses the following approaches to facilitate recovery after sepsis hospitalization: (Check all that apply; check at least one.)

- ☐ Communicating a patient's sepsis diagnosis and care plan to the patient's primary care physician

Assurance of Confidentiality: The voluntarily provided information obtained in this surveillance system that would permit identification of any individual or institution is collected with a guarantee that it will be held in strict confidence, will be used only for the purposes stated, and will not otherwise be disclosed or released without the consent of the individual, or the institution in accordance with Sections 304, 306 and 308(d) of the Public Health Service Act (42 USC 242b, 242k, and 242m(d)).
CDC 57.103 (Front) Rev. 15, v12.0

Public reporting burden of this collection of information is estimated to average 135 minutes per response, including the time for reviewing instructions, searching existing data sources, gathering, and maintaining the data needed, and completing and reviewing the collection of information. An agency may not conduct or sponsor, and a person is not required to respond to a collection of information unless it displays a currently valid OMB control number. Send comments regarding this burden estimate or any other aspect of this collection of information, including suggestions for reducing this burden to CDC, Reports Clearance Officer, 1600 Clifton Rd., MS H-21, Atlanta, GA 30333, ATTN: PRA (0920-0666).

- ☐ Providing contact information for a clinical staff at the hospital to addresses post-discharge questions and/or troubleshoot post-discharge issues
- ☐ Contacting patients within 2 days of discharge by clinical staff to follow-up on discharge instructions, symptoms, and/or issues
- ☐ Screening patients for new functional and/or cognitive impairment after sepsis and referring patients to relevant evaluation or support services
- ☐ Reconciling and optimizing medications prior to hospital discharge
- ☐ Screening patients for social vulnerability and referring to available support services as needed
- ☐ None of the above

Assurance of Confidentiality: The voluntarily provided information obtained in this surveillance system that would permit identification of any individual or institution is collected with a guarantee that it will be held in strict confidence, will be used only for the purposes stated, and will not otherwise be disclosed or released without the consent of the individual, or the institution in accordance with Sections 304, 306 and 308(d) of the Public Health Service Act (42 USC 242b, 242k, and 242m(d)).

Public reporting burden of this collection of information is estimated to average 135 minutes per response, including the time for reviewing instructions, searching existing data sources, gathering, and maintaining the data needed, and completing and reviewing the collection of information. An agency may not conduct or sponsor, and a person is not required to respond to a collection of information unless it displays a currently valid OMB control number. Send comments regarding this burden estimate or any other aspect of this collection of information, including suggestions for reducing this burden to CDC, Reports Clearance Officer, 1600 Clifton Rd., MS H-21, Atlanta, GA 30333, ATTN: PRA (0920-0666).

Sepsis Management and Practices (continued)

*67. Our facility uses the following approaches to ensure that all patients hospitalized with sepsis (or their family or caregivers), are educated on their diagnosis of sepsis, the underlying infection, and signs and symptoms of new infection or sepsis. (Check all that apply; check at least one.)

- ☐ Direct 1:1 education on sepsis from a healthcare personnel
- ☐ Written educational material about sepsis
- ☐ Pre-recorded video material about sepsis
- ☐ None of the above are used routinely

*68. Our facility tracks the following hospital sepsis metrics: (Check all that apply; check at least one.)

- ☐ Hospital sepsis epidemiology (e.g., number and characteristics of sepsis hospitalizations)
- ☐ Hospital sepsis treatment (e.g., time-to-antibiotics, type, and volume of fluid delivery)
- ☐ Hospital sepsis outcomes (e.g., mortality, length of hospitalization)
- ☐ Progress towards achieving hospital goals for sepsis treatment and/or outcomes
- ☐ Use of hospital sepsis tools (e.g., how often sepsis order-set is used)
- ☐ Usability or acceptability of hospital sepsis tools (e.g., clinician acceptance)
- ☐ Impact of hospital sepsis tools (e.g., impact on sepsis alert or order-set on treatment or outcomes)
- ☐ None of the above

*69. Describe your facility's use of manual chart review for sepsis performance evaluation and improvement: (Check one.)

- ☐ We review all sepsis hospitalizations
- ☐ We review all sepsis hospitalizations with adverse outcomes (e.g., all hospitalizations with in-hospital mortality)
- ☐ We review a sample of sepsis hospitalizations (e.g., a random sample)
- ☐ We do not complete routine chart reviews of sepsis hospitalizations

*70. Sepsis treatment and/or outcome data are reported to unit-based or service-based leadership at following frequency: (Check one)

- ☐ Continuously (e.g., a sepsis dashboard that updates in real-time)
- ☐ At least monthly
- ☐ At least quarterly
- ☐ At least annually
- ☐ Not reported or reported less often than annually

Assurance of Confidentiality: The voluntarily provided information obtained in this surveillance system that would permit identification of any individual or institution is collected with a guarantee that it will be held in strict confidence, will be used only for the purposes stated, and will not otherwise be disclosed or released without the consent of the individual, or the institution in accordance with Sections 304, 306 and 308(d) of the Public Health Service Act (42 USC 242b, 242k, and 242m(d)).
CDC 57.103 (Front) Rev. 15, v12.0

Public reporting burden of this collection of information is estimated to average 135 minutes per response, including the time for reviewing instructions, searching existing data sources, gathering, and maintaining the data needed, and completing and reviewing the collection of information. An agency may not conduct or sponsor, and a person is not required to respond to a collection of information unless it displays a currently valid OMB control number. Send comments regarding this burden estimate or any other aspect of this collection of information, including suggestions for reducing this burden to CDC, Reports Clearance Officer, 1600 Clifton Rd., MS H-21, Atlanta, GA 30333, ATTN: PRA (0920-0666).

70a. [If Q70 has one of the following answers selected: “continuously”, “at least monthly”, “at least quarterly”, or “at least annually”] Feedback data provided to clinician and/or unit-based leadership on sepsis treatment and outcomes includes the following elements at least annually: (Check all that apply; check at least one)

☐ Unit-specific or service-specific data
☐ Clinician-specific data
☐ Benchmarking or comparative data (i.e., comparison to other similar units or hospitals)
☐ Temporal trends (i.e., how treatment or outcomes have changed overtime)
☐ None of the above

Assurance of Confidentiality: The voluntarily provided information obtained in this surveillance system that would permit identification of any individual or institution is collected with a guarantee that it will be held in strict confidence, will be used only for the purposes stated, and will not otherwise be disclosed or released without the consent of the individual, or the institution in accordance with Sections 304, 306 and 308(d) of the Public Health Service Act (42 USC 242b, 242k, and 242m(d)).

Public reporting burden of this collection of information is estimated to average 135 minutes per response, including the time for reviewing instructions, searching existing data sources, gathering, and maintaining the data needed, and completing and reviewing the collection of information. An agency may not conduct or sponsor, and a person is not required to respond to a collection of information unless it displays a currently valid OMB control number. Send comments regarding this burden estimate or any other aspect of this collection of information, including suggestions for reducing this burden to CDC, Reports Clearance Officer, 1600 Clifton Rd., MS H-21, Atlanta, GA 30333, ATTN: PRA (0920-0666).

Sepsis Management and Practices (continued)

*71. Clinicians receive feedback regarding their care of specific patients with sepsis: (Check all that apply; check at least one)

- ☐ Yes, positive feedback is provided for good sepsis care
- ☐ Yes, constructive feedback is provided for areas of improvement
- ☐ Neither of the above

*72. Our facility provides education on sepsis to the following groups as part of their hiring or onboarding process: (Check all that apply; check at least one)

- ☐ APPs
- ☐ Certified nursing assistants
- ☐ Nurses
- ☐ Patient care technicians
- ☐ Physicians
- ☐ Trainees (for example, medical students, residents, nursing students)
- ☐ None of the above

*73. Our facility provides sepsis education to the following groups at least annually, for example through lectures, staff meetings, etc.: (check all that apply; check at least one)

- ☐ APPs
- ☐ Certified nursing assistants
- ☐ Nurses
- ☐ Patient care technicians
- ☐ Physicians
- ☐ None of the above

Facility Water Management Program (WMP) (Completed with input from WMP team members.)

*74. Does your facility have a water management program (WMP) to prevent the growth and transmission of *Legionella* and other opportunistic waterborne pathogens (for example, *Pseudomonas, Acinetobacter, Burkholderia, Stenotrophomonas,* nontuberculous mycobacteria, and fungi)?

☐ Yes ☐ No

74a. If Yes, who is represented on your facility WMP team? (Check all that apply):

- ☐ Hospital Epidemiologist/Infection Preventionist
- ☐ Hospital Administrator/Leadership
- ☐ Facilities Manager/Engineer
- ☐ Compliance/Safety Officer
- ☐ Risk/Quality Management Staff
- ☐ Infectious Disease Clinician

Assurance of Confidentiality: The voluntarily provided information obtained in this surveillance system that would permit identification of any individual or institution is collected with a guarantee that it will be held in strict confidence, will be used only for the purposes stated, and will not otherwise be disclosed or released without the consent of the individual, or the institution in accordance with Sections 304, 306 and 308(d) of the Public Health Service Act (42 USC 242b, 242k, and 242m(d)).

Public reporting burden of this collection of information is estimated to average 135 minutes per response, including the time for reviewing instructions, searching existing data sources, gathering, and maintaining the data needed, and completing and reviewing the collection of information. An agency may not conduct or sponsor, and a person is not required to respond to a collection of information unless it displays a currently valid OMB control number. Send comments regarding this burden estimate or any other aspect of this collection of information, including suggestions for reducing this burden to CDC, Reports Clearance Officer, 1600 Clifton Rd., MS H-21, Atlanta, GA 30333, ATTN: PRA (0920-0666).

☐ Maintenance Staff
☐ Equipment/Chemical Acquisition/Supplier
☐ Environmental Services
☐ Consultant
☐ Laboratory Staff/Leadership
☐ Other (specify): ____________________

Assurance of Confidentiality: The voluntarily provided information obtained in this surveillance system that would permit identification of any individual or institution is collected with a guarantee that it will be held in strict confidence, will be used only for the purposes stated, and will not otherwise be disclosed or released without the consent of the individual, or the institution in accordance with Sections 304, 306 and 308(d) of the Public Health Service Act (42 USC 242b, 242k, and 242m(d)).
CDC 57.103 (Front) Rev. 15, v12.0

Public reporting burden of this collection of information is estimated to average 135 minutes per response, including the time for reviewing instructions, searching existing data sources, gathering, and maintaining the data needed, and completing and reviewing the collection of information. An agency may not conduct or sponsor, and a person is not required to respond to a collection of information unless it displays a currently valid OMB control number. Send comments regarding this burden estimate or any other aspect of this collection of information, including suggestions for reducing this burden to CDC, Reports Clearance Officer, 1600 Clifton Rd., MS H-21, Atlanta, GA 30333, ATTN: PRA (0920-0666).

Facility Water Management Program (WMP) (continued)

*75. Has your facility ever conducted an environmental assessment to identify where *Legionella* and other opportunistic waterborne pathogens could grow and spread in the facility water system (for example, piping infrastructure)? This may include a description of building water systems using text or basic diagrams that map all water supply sources, treatment systems, processing steps, control measures, and end-use points.

☐ Yes ☐ No

75a. If Yes, when was the most recent assessment conducted? (Check one)

☐ Within the most recent year (≤ 1 year ago) ☐ Between 1 and 3 years ago (> 1 year and ≤ 3 years) ☐ More than 3 years ago (> 3 years)

*76. Has your facility ever conducted a water infection control risk assessment (WICRA) to evaluate water sources, modes of transmission, patient susceptibility, patient exposure, and/or program preparedness? An example WICRA tool can be accessed at https://www.cdc.gov/hai/pdfs/prevent/water-assessment-tool-508.pdf

☐ Yes ☐ No

76a. If Yes, when was the most recent assessment conducted? (Check one)

☐ Within the most recent year (≤ 1 year ago) ☐ Between 1 and 3 years ago (> 1 year and ≤ 3 years) ☐ More than 3 years ago (> 3 years)

*77. Does your facility regularly monitor the following parameters in the building water system(s)?

Disinfectant (such as residual chlorine): ☐ Yes ☐ No

77a. If Yes, does your facility have a plan for corrective actions when disinfectant(s) are not within acceptable limits as determined by the water management program? ☐ Yes ☐ No

77b. If Yes, where and how frequently does your facility monitor disinfectant(s)? (Check all that apply)

	Entry Points	Cold Potable Water Storage Tank(s)	Hot Potable Water Storage Tank(s)	Hot Water Supply	Hot Water Return	Representative Locations Throughout Cold Potable Building Water System(s)	Representative Locations Throughout Hot Potable Building Water System(s)	Other (specify): ________
Daily	☐	☐	☐	☐	☐	☐	☐	☐
Weekly	☐	☐	☐	☐	☐	☐	☐	☐
Monthly	☐	☐	☐	☐	☐	☐	☐	☐

Assurance of Confidentiality: The voluntarily provided information obtained in this surveillance system that would permit identification of any individual or institution is collected with a guarantee that it will be held in strict confidence, will be used only for the purposes stated, and will not otherwise be disclosed or released without the consent of the individual, or the institution in accordance with Sections 304, 306 and 308(d) of the Public Health Service Act (42 USC 242b, 242k, and 242m(d)).
CDC 57.103 (Front) Rev. 15, v12.0

Public reporting burden of this collection of information is estimated to average 135 minutes per response, including the time for reviewing instructions, searching existing data sources, gathering, and maintaining the data needed, and completing and reviewing the collection of information. An agency may not conduct or sponsor, and a person is not required to respond to a collection of information unless it displays a currently valid OMB control number. Send comments regarding this burden estimate or any other aspect of this collection of information, including suggestions for reducing this burden to CDC, Reports Clearance Officer, 1600 Clifton Rd., MS H-21, Atlanta, GA 30333, ATTN: PRA (0920-0666).

Quarterly	☐	☐	☐	☐	☐	☐	☐	☐
Annually	☐	☐	☐	☐	☐	☐	☐	☐
Other (specify):__________	☐	☐	☐	☐	☐	☐	☐	☐

Facility Water Management Program (WMP) (continued)

Water Temperature: ☐ Yes ☐ No

77c. If Yes, does your facility have a plan for corrective actions when water temperatures are not within acceptable limits as determined by the water management program? ☐ Yes ☐ No

77d. If Yes, where and how frequently does your facility monitor water temperature? (check all that apply)

	Entry Points	Cold Potable Water Storage Tank(s)	Hot Potable Water Storage Tank(s)	Hot Water Supply	Hot Water Return	Representative Locations Throughout Cold Potable Building Water System(s)	Representative Locations Throughout Hot Potable Building Water System(s)	Other (specify): ________
Daily	☐	☐	☐	☐	☐	☐	☐	☐
Weekly	☐	☐	☐	☐	☐	☐	☐	☐
Monthly	☐	☐	☐	☐	☐	☐	☐	☐
Quarterly	☐	☐	☐	☐	☐	☐	☐	☐
Annually	☐	☐	☐	☐	☐	☐	☐	☐
Other (specify):__________	☐	☐	☐	☐	☐	☐	☐	☐

Water pH: ☐ Yes ☐ No

77e. If Yes, does your facility have a plan for corrective actions when water pH is not within acceptable limits as determined by the water management program? ☐ Yes ☐ No

77f. If Yes, where and how frequently does your facility monitor water pH? (check all that apply)

	Entry Points	Cold Potable Water Storage Tank(s)	Hot Potable Water Storage Tank(s)	Hot Water Supply	Hot Water Return	Representative Locations Throughout Cold Potable Building Water System(s)	Representative Locations Throughout Hot Potable Building Water System(s)	Other (specify): ________

Assurance of Confidentiality: The voluntarily provided information obtained in this surveillance system that would permit identification of any individual or institution is collected with a guarantee that it will be held in strict confidence, will be used only for the purposes stated, and will not otherwise be disclosed or released without the consent of the individual, or the institution in accordance with Sections 304, 306 and 308(d) of the Public Health Service Act (42 USC 242b, 242k, and 242m(d)).

Public reporting burden of this collection of information is estimated to average 135 minutes per response, including the time for reviewing instructions, searching existing data sources, gathering, and maintaining the data needed, and completing and reviewing the collection of information. An agency may not conduct or sponsor, and a person is not required to respond to a collection of information unless it displays a currently valid OMB control number. Send comments regarding this burden estimate or any other aspect of this collection of information, including suggestions for reducing this burden to CDC, Reports Clearance Officer, 1600 Clifton Rd., MS H-21, Atlanta, GA 30333, ATTN: PRA (0920-0666).

Daily	☐	☐	☐	☐	☐	☐	☐	☐
Weekly	☐	☐	☐	☐	☐	☐	☐	☐
Monthly	☐	☐	☐	☐	☐	☐	☐	☐
Quarterly	☐	☐	☐	☐	☐	☐	☐	☐
Annually	☐	☐	☐	☐	☐	☐	☐	☐
Other (specify):__________	☐	☐	☐	☐	☐	☐	☐	☐

Heterotrophic plate count (HPC) testing: ☐ Yes ☐ No

77g. If Yes, does your facility have a plan for corrective actions when heterotrophic plate counts are not within acceptable limits as determined by the water management program? ☐ Yes ☐ No

77h. If Yes, where and how frequently does your facility perform HPC testing? (check all that apply)

Assurance of Confidentiality: The voluntarily provided information obtained in this surveillance system that would permit identification of any individual or institution is collected with a guarantee that it will be held in strict confidence, will be used only for the purposes stated, and will not otherwise be disclosed or released without the consent of the individual, or the institution in accordance with Sections 304, 306 and 308(d) of the Public Health Service Act (42 USC 242b, 242k, and 242m(d)).
CDC 57.103 (Front) Rev. 15, v12.0

Public reporting burden of this collection of information is estimated to average 135 minutes per response, including the time for reviewing instructions, searching existing data sources, gathering, and maintaining the data needed, and completing and reviewing the collection of information. An agency may not conduct or sponsor, and a person is not required to respond to a collection of information unless it displays a currently valid OMB control number. Send comments regarding this burden estimate or any other aspect of this collection of information, including suggestions for reducing this burden to CDC, Reports Clearance Officer, 1600 Clifton Rd., MS H-21, Atlanta, GA 30333, ATTN: PRA (0920-0666).

Facility Water Management Program (WMP) (continued)

	Entry Points	Cold Potable Water Storage Tank(s)	Hot Potable Water Storage Tank(s)	Hot Water Supply	Hot Water Return	Representative Locations Throughout Cold Potable Building Water System(s)	Representative Locations Throughout Hot Potable Building Water System(s)	Other (specify): ________
Daily	☐	☐	☐	☐	☐	☐	☐	☐
Weekly	☐	☐	☐	☐	☐	☐	☐	☐
Monthly	☐	☐	☐	☐	☐	☐	☐	☐
Quarterly	☐	☐	☐	☐	☐	☐	☐	☐
Annually	☐	☐	☐	☐	☐	☐	☐	☐
Other (specify):__________	☐	☐	☐	☐	☐	☐	☐	☐

Specific environmental *Legionella* testing: ☐ Yes ☐ No

77i. If Yes, does your facility have a plan for corrective actions when environmental tests for *Legionella* are not within acceptable limits as determined by the water management program? ☐ Yes ☐ No

77j. If Yes, where an how frequently does your facility perform *Legionella* testing? (check all that apply)

	Entry Points	Cold Potable Water Storage Tank(s)	Hot Potable Water Storage Tank(s)	Hot Water Supply	Hot Water Return	Representative Locations Throughout Cold Potable Building Water System(s)	Representative Locations Throughout Hot Potable Building Water System(s)	Other (specify): ________
Daily	☐	☐	☐	☐	☐	☐	☐	☐
Weekly	☐	☐	☐	☐	☐	☐	☐	☐
Monthly	☐	☐	☐	☐	☐	☐	☐	☐
Quarterly	☐	☐	☐	☐	☐	☐	☐	☐
Annually	☐	☐	☐	☐	☐	☐	☐	☐
Other (specify):__________	☐	☐	☐	☐	☐	☐	☐	☐

Specific environmental *Pseudomonas* testing: ☐ Yes ☐ No

77k. If Yes, does your facility have a plan for corrective actions when environmental tests for *Pseudomonas* are not within acceptable limits as determined by the water management program?

Assurance of Confidentiality: The voluntarily provided information obtained in this surveillance system that would permit identification of any individual or institution is collected with a guarantee that it will be held in strict confidence, will be used only for the purposes stated, and will not otherwise be disclosed or released without the consent of the individual, or the institution in accordance with Sections 304, 306 and 308(d) of the Public Health Service Act (42 USC 242b, 242k, and 242m(d)).

Public reporting burden of this collection of information is estimated to average 135 minutes per response, including the time for reviewing instructions, searching existing data sources, gathering, and maintaining the data needed, and completing and reviewing the collection of information. An agency may not conduct or sponsor, and a person is not required to respond to a collection of information unless it displays a currently valid OMB control number. Send comments regarding this burden estimate or any other aspect of this collection of information, including suggestions for reducing this burden to CDC, Reports Clearance Officer, 1600 Clifton Rd., MS H-21, Atlanta, GA 30333, ATTN: PRA (0920-0666).

☐ Yes ☐ No

77l. If Yes, where an how frequently does your facility perform *Pseudomonas* testing? (check all that apply)

Assurance of Confidentiality: The voluntarily provided information obtained in this surveillance system that would permit identification of any individual or institution is collected with a guarantee that it will be held in strict confidence, will be used only for the purposes stated, and will not otherwise be disclosed or released without the consent of the individual, or the institution in accordance with Sections 304, 306 and 308(d) of the Public Health Service Act (42 USC 242b, 242k, and 242m(d)).
CDC 57.103 (Front) Rev. 15, v12.0

Public reporting burden of this collection of information is estimated to average 135 minutes per response, including the time for reviewing instructions, searching existing data sources, gathering, and maintaining the data needed, and completing and reviewing the collection of information. An agency may not conduct or sponsor, and a person is not required to respond to a collection of information unless it displays a currently valid OMB control number. Send comments regarding this burden estimate or any other aspect of this collection of information, including suggestions for reducing this burden to CDC, Reports Clearance Officer, 1600 Clifton Rd., MS H-21, Atlanta, GA 30333, ATTN: PRA (0920-0666).

Facility Water Management Program (WMP) (continued)

	Entry Points	Cold Potable Water Storage Tank(s)	Hot Potable Water Storage Tank(s)	Hot Water Supply	Hot Water Return	Representative Locations Throughout Cold Potable Building Water System(s)	Representative Locations Throughout Hot Potable Building Water System(s)	Other (specify): ________
Daily	☐	☐	☐	☐	☐	☐	☐	☐
Weekly	☐	☐	☐	☐	☐	☐	☐	☐
Monthly	☐	☐	☐	☐	☐	☐	☐	☐
Quarterly	☐	☐	☐	☐	☐	☐	☐	☐
Annually	☐	☐	☐	☐	☐	☐	☐	☐
Other (specify):__________	☐	☐	☐	☐	☐	☐	☐	☐

*78. Does your facility water management program address measures to prevent transmission of pathogens from wastewater premise plumbing to patients?

☐ Yes ☐ No ☐ N/A, my facility does not have a water management program

Assurance of Confidentiality: The voluntarily provided information obtained in this surveillance system that would permit identification of any individual or institution is collected with a guarantee that it will be held in strict confidence, will be used only for the purposes stated, and will not otherwise be disclosed or released without the consent of the individual, or the institution in accordance with Sections 304, 306 and 308(d) of the Public Health Service Act (42 USC 242b, 242k, and 242m(d)).
CDC 57.103 (Front) Rev. 15, v12.0

Public reporting burden of this collection of information is estimated to average 135 minutes per response, including the time for reviewing instructions, searching existing data sources, gathering, and maintaining the data needed, and completing and reviewing the collection of information. An agency may not conduct or sponsor, and a person is not required to respond to a collection of information unless it displays a currently valid OMB control number. Send comments regarding this burden estimate or any other aspect of this collection of information, including suggestions for reducing this burden to CDC, Reports Clearance Officer, 1600 Clifton Rd., MS H-21, Atlanta, GA 30333, ATTN: PRA (0920-0666).
